# Association between maternal adiposity measures and infant health outcomes: A systematic review and meta‐analysis

**DOI:** 10.1111/obr.13491

**Published:** 2022-07-08

**Authors:** Giang Nguyen, Louise Hayes, Lem Ngongalah, Theophile Bigirumurame, Laura Gaudet, Adefisayo Odeniyi, Angela Flynn, Lisa Crowe, Becky Skidmore, Alexandre Simon, Vikki Smith, Nicola Heslehurst

**Affiliations:** ^1^ Population Health Sciences Institute Newcastle University Newcastle upon Tyne UK; ^2^ Department of Obstetrics and Gynaecology Queen's University Kingston Ontario Canada; ^3^ Department of Nutritional Sciences King's College London London UK; ^4^ Independent Information Specialist Ottawa Ontario Canada; ^5^ Department of Obstetrics and Gynaecology University of Ottawa Ottawa Ontario Canada; ^6^ Nursing, Midwifery & Health Northumbria University Newcastle upon Tyne UK

**Keywords:** adiposity, infant, obesity, pregnancy

## Abstract

Maternal obesity increases risks of adverse fetal and infant outcomes. Guidelines use body mass index to diagnose maternal obesity. Evidence suggests body fat distribution might better predict individual risk, but there is a lack of robust evidence during pregnancy. We explored associations between maternal adiposity and infant health. Searches included six databases, references, citations, and contacting authors. Screening and quality assessment were carried out by two authors independently. Random effects meta‐analysis and narrative synthesis were conducted. We included 34 studies (*n* = 40,143 pregnancies). Meta‐analysis showed a significant association between maternal fat‐free mass and birthweight (average effect [AE] 18.07 g, 95%CI 12.75, 23.38) but not fat mass (AE 8.76 g, 95%CI −4.84, 22.36). Women with macrosomic infants had higher waist circumference than controls (mean difference 4.93 cm, 95% confidence interval [CI] 1.05, 8.82). There was no significant association between subcutaneous fat and large for gestational age (odds ratio 1.06 95% CI 0.91, 1.25). Waist‐to‐hip ratio, neck circumference, skinfolds, and visceral fat were significantly associated with several infant outcomes including small for gestational age, preterm delivery, neonatal morbidity, and mortality, although meta‐analysis was not possible for these variables. Our findings suggest that some measures of maternal adiposity may be useful for risk prediction of infant outcomes. Individual participant data meta‐analysis could overcome some limitations in our ability to pool published data.

**Abbreviations**
BMI: Body mass index
CI: confidence interval
FM: Fat mass
FFM: Fat free mass
IUGR: Intrauterine growth restriction
LGA: large for gestational age
MD: mean difference
MOOSE: Meta‐analysis of observational studies
NICU: Neonatal intensive care unit
OR: Odds ratio
SGA: small for gestational age
SFT: Skinfold thickness
WC: Waist circumference
WHR: Waist‐to‐hip ratio

## 1 INTRODUCTION

Maternal obesity is arguably the leading challenge for pregnancy‐related clinical practice.^1^ It is a risk factor for several adverse maternal, fetal, and infant outcomes.^2^ There is a wealth of evidence demonstrating the association between maternal obesity, measured using body mass index (BMI), and increased risk of immediate adverse outcomes for the fetus and infant (hereon called “infant”), as well as lifelong health and well‐being.^2^, ^3^, ^4^ Immediate infant health outcomes include abnormal fetal growth,^5^ congenital anomalies,^6^ preterm birth,^7^ small‐ and large‐ for gestational age (SGA and LGA) infants,^8^, ^9^ infant morbidity and mortality, and consequently, increased risk of childhood obesity^10^ with associated long‐term complications such as type 2 diabetes.^11^

It is well accepted that the in‐utero environment critically influences both short‐ and long‐term health outcomes of infants,^12^ making maternal obesity a priority research area relating to optimizing child health. Newborns exposed to insufficient or excess maternal nutrition are more likely to have had abnormal in‐utero growth, including both fetal growth restriction and fetal overgrowth. This abnormal fetal growth contributes to both adverse birth outcomes and neonatal morbidity, including hypoglycemia, hypothermia, and neonatal intensive care unit (NICU) admission. The Barker hypothesis maintains that adverse nutrition during pregnancy increases the life‐long risk of metabolic syndrome in infants, including obesity, diabetes, hypertension, hyperlipidemia, coronary artery disease, and stroke.^12^ A suboptimal in‐utero environment prompts epigenetic modification of genes critical for metabolic programming of the fetus.^13^ Subsequent transgenerational transmission of these modifications then increases risks for generations to come.^14^ Further, evidence is accumulating that a healthy microbiome confers positive metabolic programming of infants and children.^15^ It is suggested that maternal obesity influences the microbial colonization in intrauterine environment.^16^ Since the microbiome of the infant is largely inherited from the mother, modification could result in a more optimal intra‐uterine environment and improve both immediate postnatal and longer‐term health of infants.

Diet and physical activity interventions, which aim to reduce the infant health risks associated with maternal obesity, have been inconsistent in their findings to date. For example, while meta‐analysis shows a general pattern of interventions reducing the risk of high birthweight outcomes, there is generally a lack of significant difference between intervention and control arms of trials.^17^ As there is clear evidence of the potential benefits of changing the in‐utero environment to improve infant outcomes, perhaps the lack of effect to date could be due to the current use of maternal BMI in identifying which pregnancies might be at a higher risk. BMI is used in international guidelines to diagnose an individual's weight status (i.e., obesity) for risk stratification of pregnant women with the aim of improving maternal and infant health outcomes.^18^, ^19^, ^20^ BMI is a useful tool to identify population trends in obesity‐related disease.^18^ However, it is well established in non‐pregnancy literature that BMI has high specificity but low sensitivity to detect excess adiposity in individuals and fails to identify half of people with excess body fat.^21^, ^22^ Alternative measures of body fat distribution have been more successful in predicting individual risk. For example, waist circumference (WC) has been used for a number of years to assess abdominal obesity as an alternative to, or alongside, BMI, as it is highly correlated with visceral fat.^21^ In pregnancy, it has been suggested that early‐pregnancy abdominal adiposity is associated with maternal metabolic consequences and could be a better marker of metabolic risks and fetal size than BMI alone.^23^, ^24^, ^25^ The evidence base from risk prediction models (in non‐pregnant populations) suggests that the use of WC as a continuous variable, adjusted for BMI, works better than BMI alone to identify individuals with a high‐risk obesity phenotype.^26^ Other measures, such as early pregnancy waist‐to‐hip ratio (WHR) and subcutaneous fat thickness, have also been suggested to better predict pregnancy outcomes than BMI, as these may be more reflective of abdominal obesity.^25^, ^27^, ^28^ This evidence highlights the importance of better understanding of adiposity‐related risk in efforts to improve infant health in short and long terms. However, there is a lack of robust evidence relating to maternal adiposity measures and infant health‐related outcomes and whether these measures work better than BMI to predict risk. This systematic review and meta‐analysis aimed to identify measures of early pregnancy adiposity that are associated with infant health outcomes which may be candidate alternative measures to the current use of BMI.

## 2 METHODS

This systematic review was conducted alongside a systematic review and meta‐analysis of maternal outcomes, and the details of the searches have been reported in full elsewhere.^29^ The methods are also summarized here, with details on the search and amendments to the inclusion criteria specific to the aim of this paper. The systematic review was registered on PROSPERO (CRD42017064464).^30^ Both observational cohort and cross‐sectional studies were included; therefore, the meta‐analysis of observational studies (MOOSE) guidelines were followed.^31^

The search strategy was derived by an experienced information specialist using the concepts “Pregnancy,” “Adiposity,” “Prediction/Risk,” and “Outcomes” and was peer reviewed by another experienced information specialist using the PRESS checklist.^32^ Databases searched were MEDLINE, EMBASE, PsycINFO, CINAHL (EBSCO), JBI Database, and the Cochrane Library. Full details of the search terms are reported elsewhere.^29^ Following MOOSE recommendations for comprehensive searches to include sources supplementary to databases,^31^ all reference lists of included studies were hand searched and citation searches were carried out using the Google Scholar “cited by” feature. Any new studies which met the inclusion criteria were also reference and citation searched. Authors were contacted when additional information was required for analyses (Table S2). Database searches were completed in April 2021, citation and reference list searches in June 2021, and contacting authors in January 2022.

Inclusion criteria were based on PECOS.^33^ The population (P) were singleton pregnancies, with any exposures (E) of pre‐ or early‐pregnancy measures of high adiposity (measured ≤20 weeks' gestation). We included prospective or retrospective observational peer‐reviewed studies, including cohort, case control, and cross‐sectional studies (S) with a comparison group of low adiposity (C). We included any pregnancy outcomes relating to infant health (O). We excluded RCTs and studies which were restricted to sub‐populations (e.g., adolescents and pre‐existing type 2 diabetes), with the exception of BMI to explore associations across a range of BMIs. No country, language, or date restrictions were applied at the screening stage. Screening results are reported using the PRISMA statement.^34^

The data were extracted by one author and validated by a second (NH, LN, AO, AF, LH, AS, LC, and VS). A standardized protocol was used, which included the study context, design and conduct, the adiposity measures, infant outcomes, and results reported. Two authors independently carried out Newcastle‐Ottawa quality assessments for cohort and case control studies to assess information bias, selection bias, and confounding^35^ (Table S1). Conflicts in data extraction or quality assessment were resolved by discussion or a third author. We assessed all included studies for duplicate publication of the same population. Two studies^36^, ^37^ reported data from the same cohort. There was overlapping data relating to fat‐free mass and birthweight, and newborn anthropometry (head circumference, crown–heel length) reported by both papers; therefore, we excluded these data reported by one of the papers^36^ from the analysis.

Methods of analysis have been reported elsewhere.^29^ Meta‐analysis was carried out when three or more studies reported data suitable for pooling on the associations between early pregnancy adiposity measure and infant outcomes. All studies were examined for quality prior to meta‐analysis. To be eligible for meta‐analysis, the combinations of adiposity exposures and infant outcomes, and type of data (e.g., odds ratio [OR] and mean difference [MD]) needed to be similar enough to justify pooling. Summary of ORs, MDs, and average treatment effects (AE) were calculated using the random effects model by restricted maximum likelihood.^38^, ^39^ The *I* ^*2*^ statistic was used to assess the heterogeneity among studies,^40^ with a threshold of >75% representing significant heterogeneity.^41^ Due to the small number of studies in each meta‐analysis, we were not able to perform meta‐regression, sensitivity analysis, sub‐group analysis, or tests for publication bias^42^, ^43^ as planned in our PROSPERO protocol.^30^ The statistical analyses were conducted using *metafor* ^44^ packages for R version 4.0.4. A narrative synthesis was carried using recommendations by Popay *et al*.^45^ when meta‐analysis was not possible. Data from each study were tabulated and grouped according to the outcome being reported, sub‐grouped by the adiposity exposure, and patterns were described.

## 3 RESULTS

**FIGURE 1:**
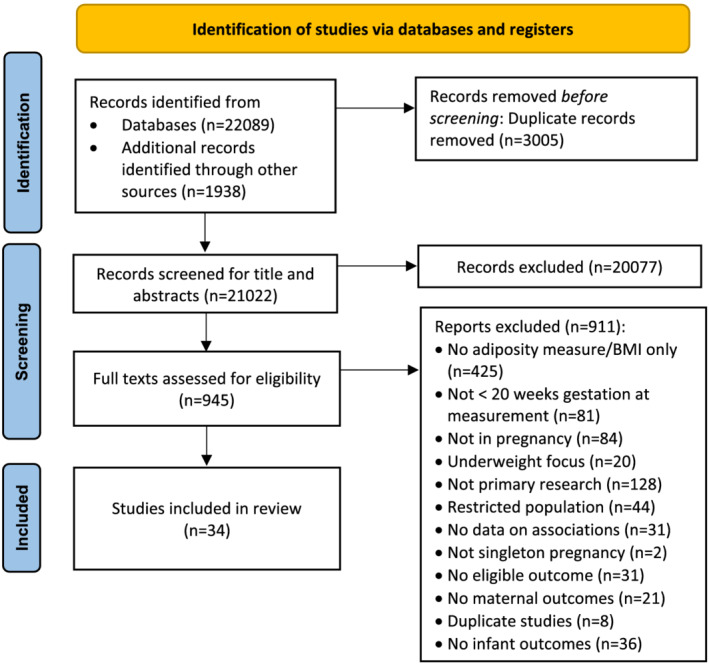
PRISMA Flow‐chart of the study selection process. Adapted from: Page et al^34^

There were 24,027 studies identified following removal of duplicates, and 945 of these proceeded to the full text screening stage. Thirty‐four studies^23^, ^24^, ^25^, ^28^, ^36^, ^37^, ^46^, ^47^, ^48^, ^49^, ^50^, ^51^, ^52^, ^53^, ^54^, ^55^, ^56^, ^57^, ^58^, ^59^, ^60^, ^61^, ^62^, ^63^, ^64^, ^65^, ^66^, ^67^, ^68^, ^69^, ^70^, ^71^, ^72^, ^73^ met the inclusion criteria (Figure 1); 33 were cohort studies, and one was a case control study. Studies were published between 1995 and 2021, with the majority (*n* = 30, 88.2%) published between 2011 and 2021. Sample sizes ranged from 45 to 6687 pregnancies, with a pooled sample of 40,143 (Table S3). Study settings were in Europe (*n* = 11; United Kingdom *n* = 3, Ireland *n* = 3, Italy *n* = 1, Finland *n* = 1, Poland *n* = 1, Denmark *n* = 1, Sweden *n* = 1), Asia (*n* = 9; China *n* = 2, India *n* = 2, Iran *n* = 2, Turkey *n* = 1, Indonesia *n* = 1, Vietnam *n* = 1), North America (*n* = 9; United States of America *n* = 4, Canada *n* = 3, Jamaica *n* = 2), South/Central America (*n* = 3; Argentina *n* = 1, Brazil *n* = 1, Mexico *n* = 1), and Australia (*n* = 2) (Table S3). Two studies used BMI as part of their inclusion criteria: one included only women with an obese BMI (≥30.0 kg/m^2^),^48^ and one included women with a BMI between 18.5 and 35 kg/m^2^.^50^

Early pregnancy WC was the most frequently reported adiposity measure (*n* = 11 studies), followed by WHR and measures of fat mass (FM) (*n* = 9 each); fat‐free mass (FFM) (*n* = 8); visceral fat (*n* = 7); arm circumference (*n* = 4); subcutaneous fat (*n* = 3); skinfold thickness (SFT) (*n* = 3); visceral‐to‐subcutaneous fat ratio (*n* = 2); and *n* = 1 each for hip circumference, neck circumference, calf circumference, total body water, self‐reported body shape, visceral adiposity index, and combination of visceral fat and subcutaneous fat (Tables S3 and S4). The majority of outcome data reported were for infant birthweight in gram or kilogram (*n* = 16 studies), followed by LGA or macrosomia (*n* = 14); low birthweight (<2500 g), SGA or intra‐uterine growth restriction (IUGR) (*n* = 7); fetal growth and infant anthropometry at birth (*n* = 7); gestational age at delivery (*n* = 5); pregnancy loss (including spontaneous abortion, missed abortion, and spontaneous miscarriage and vesicular mole; *n* = 3); and neonatal morbidity (including NICU admission, low Apgar at 1 min, neonatal jaundice, and neonatal respiratory distress; *n* = 2) (Tables S3 and S4). Three studies^23^, ^52^, ^59^ reported composite pregnancy outcomes including both maternal and infant outcomes, which have already been reported in accompanying maternal outcomes paper,^29^ and these composite outcomes are therefore not reported in this paper.

The quality score of studies ranged from four (medium quality) to eight (high quality) (Table S5A, B). Twenty‐five cohort studies were rated as high quality, eight were rated as medium quality, and no studies were rated low quality (Table S5A). Cohort studies consistently scored highly across all assessment criteria (>70%), where adequate length of follow‐up (Q6) was met by all included cohort studies (100%), and the lowest scoring item was adequacy of follow‐up (Q7) (73%). The case control study had a score of eight (high quality) (Table S5B).

## 4 BIRTHWEIGHT

There were 16 studies^37^, ^47^, ^48^, ^49^, ^52^, ^54^, ^56^, ^57^, ^58^, ^62^, ^64^, ^70^, ^71^, ^72^, ^73^, ^74^ reporting data for maternal circumference measures (WC, arm, neck, hip, and calf circumference), ratios (WHR, visceral fat to subcutaneous fat [VAT:SAT]), fat/mass type (visceral fat, subcutaneous fat, FM, FFM), SFT (tricep, bicep, subscapular, suprailiac, sum of skinfolds), and combination of visceral adipose tissue (VAT) thickness and subcutaneous adipose tissue (SAT) thickness (VAT + SAT) (Table S6). Meta‐analysis was possible for FM and FFM. Additional birthweight data, which could not be pooled in meta‐analysis, were reported for maternal circumference measures (waist, arm, calf, hip), ratios (WHR and VAT:SAT), type of fat/mass (visceral and subcutaneous fat), and combination of VAT + SAT and SFT (Table S6). While there were some inconsistencies with statistical significance, there was an overall pattern for positive associations between birthweight and maternal FM, FFM, WHR, WC, visceral fat, VAT:SAT, VAT + SAT, and hip circumference. Associations between birthweight and the other maternal adiposity measures showed conflicting results. An overview of the meta‐analysis and narrative synthesis for birthweight is presented for each maternal adiposity exposure.

### 4.1 Maternal FM and birthweight

**FIGURE 2:**
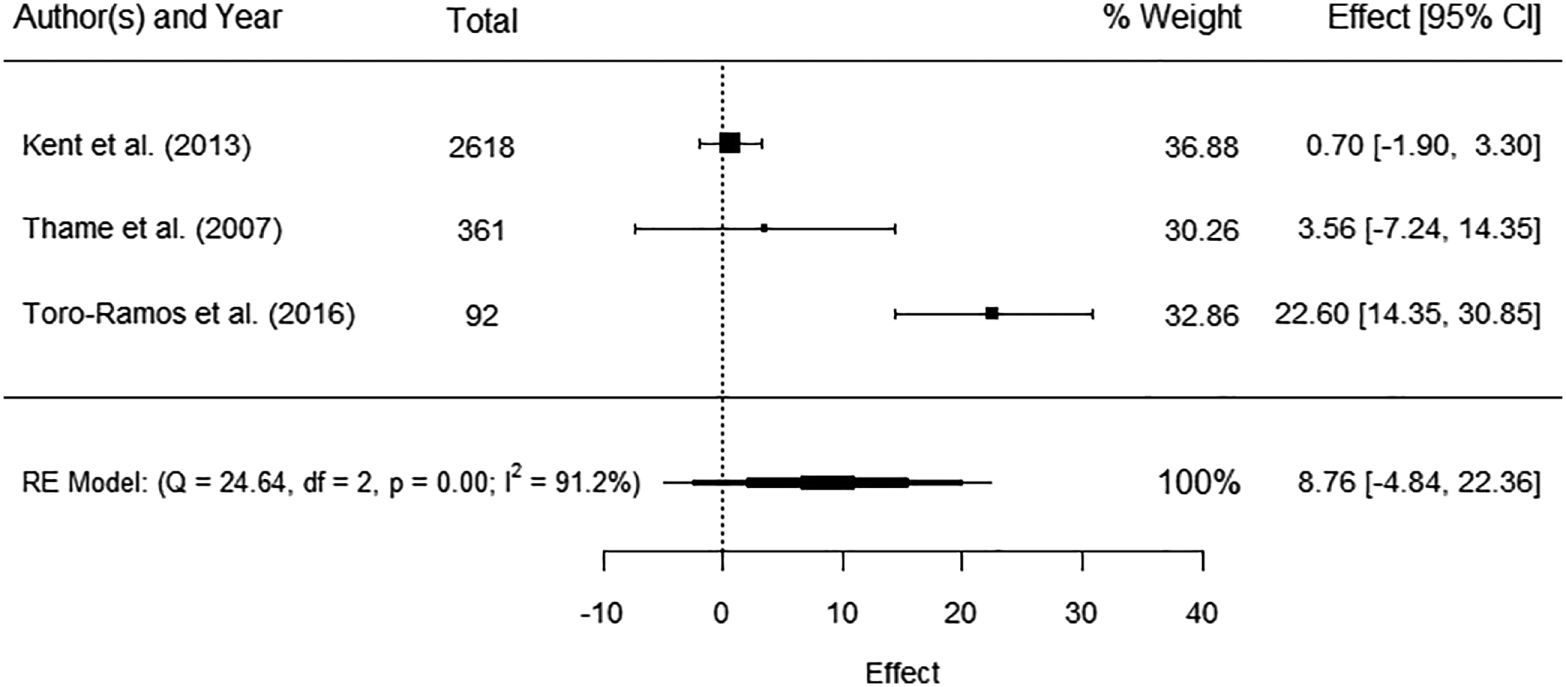
Meta‐analysis of the association between maternal fat mass (in kilograms) and birthweight (kg). Total sample size (*n* = 3071), CI – confidence interval, RE—random effect

Five studies^37^, ^58^, ^60^, ^71^, ^73^ reported maternal FM. Three^37^, ^58^, ^71^ reported FM in kilograms and were pooled in a meta‐analysis that showed a positive but non‐significant association between maternal FM and birthweight (AE 8.76 g, 95% CI −4.84, 22.36) with significant heterogeneity (*I* ^*2*^ 91.2%) (Figure 2). The two studies that could not be included in the meta‐analysis reported a significant positive correlation between maternal body fat percentage and birthweight (*r* 0.23, *p* < 0.0001)^60^ and a significant prediction of 124 g greater birthweight per 1 standard deviation (SD) increase in maternal mid‐upper arm fat area.^73^

### 4.2 Maternal FFM, muscle mass, and birthweight

**FIGURE 3:**
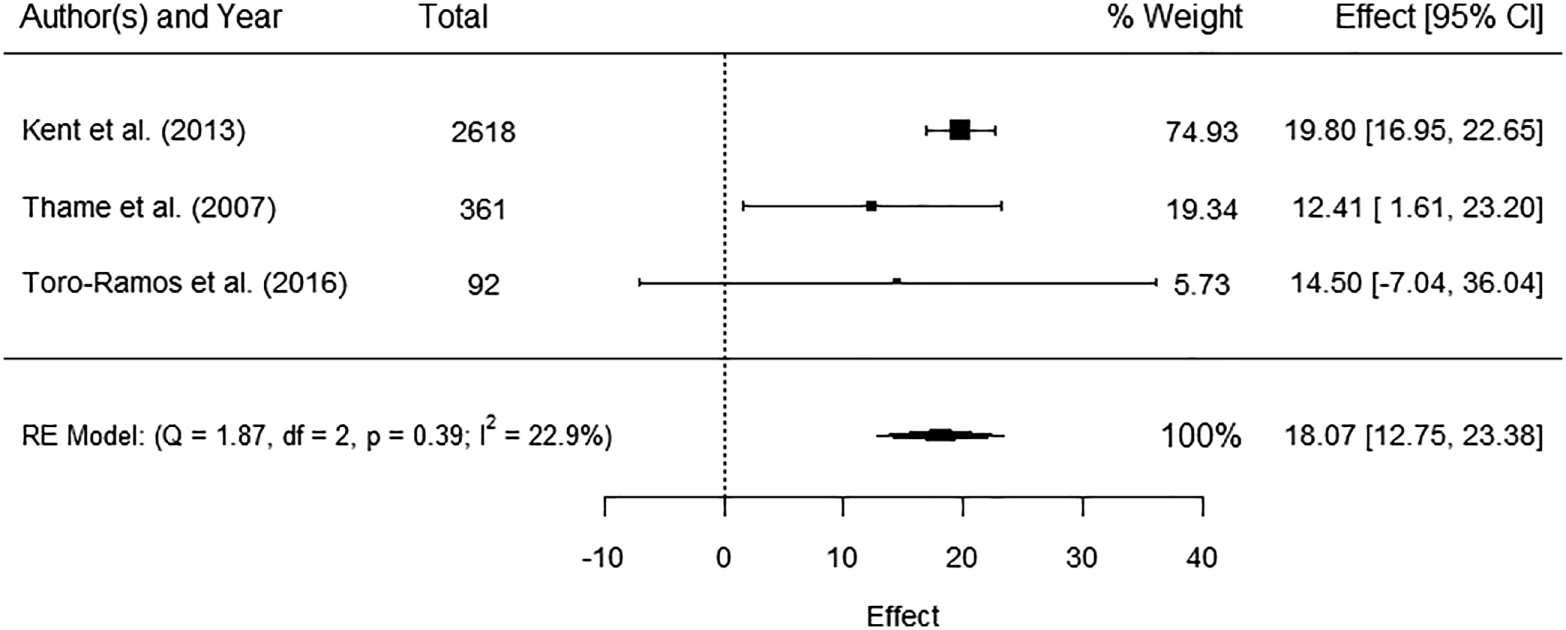
Meta‐analysis of the association between maternal fat free mass (in kilograms) and birthweight (kg). Total sample size (*n* = 3071), CI – confidence interval, RE—random effect

Three studies reported data for maternal FFM and birthweight^37^, ^58^, ^71^, ^73^ and could be pooled in a meta‐analysis. There was a significant positive association (AE 18.07 g, 95% CI 12.75, 23.38) with no significant heterogeneity (*I* ^*2*^ 22.9%) (Figure 3). One study^73^ additionally reported positive but no significant association between birthweight and maternal mid‐upper arm muscle.

### 4.3 Circumferences and birthweight

Five studies reported data for maternal WC.^52^, ^54^, ^57^, ^64^, ^70^ Three reported significant positive correlations,^54^, ^57^, ^64^ one^52^ found significantly increased mean birthweight among women with high maternal WC (>80 cm), whereas one reported no significant association.^70^ Three studies^47^, ^72^, ^73^ reported data for maternal arm circumference. One^72^ reported a significant but weak positive correlation with maternal upper arm circumference (*r* 0.271, *p* < 0.001), whereas two reported non‐significant conflicting directions of association (*r* 0.19, *p* value not reported,^47^, ^73^ and standardized estimate −5, 95% CI −108, 97^73^). One study^57^ reported a significant positive correlation between birthweight percentile and maternal hip circumference (*r* 0.32, *p* < 0.05), while one^73^ did not find any association with maternal calf circumference (standardized estimate 4, 95% CI −78, 86) (Table S6).

### 4.4 Ratios and birthweight

Three studies^49^, ^57^, ^64^ reported data for maternal WHR. Two^49^, ^64^ reported significant associations including a 0.1 unit increase in maternal WHR predicting 120 g greater newborn weight,^49^ and a positive correlation (*r* 0.6, *p* < 0.01).^64^ The third study^57^ reported positive but no significant correlation (*r* 0.01, *p* = 0.97). Two studies^57^, ^62^ reported data for birthweight and ratio of VAT:SAT, and both showed positive but non‐significant associations (adjusted beta coefficient 7.2, 95%CI −2.4, 16.8^62^ and *r* 0.01, *p* = 0.97,^57^ respectively).

### 4.5 Type of fat/mass and birthweight

Two studies^57^, ^62^ reported data for continuous measures of maternal subcutaneous fat thickness; one^57^ showed a significant positive correlation (*r* = 0.34 *p* < 0.05), while one^62^ showed no significant association (adjusted β −0.7, 95%CI −15.4, 13.9) (Table S6). Four studies^48^, ^56^, ^57^, ^62^ reported data for maternal visceral fat measures, and three showed positive significant associations. One study^48^ reported a significant but weak correlation (*r* 0.17, *p* = 0.002). A second^62^ reported a 5‐mm increase in visceral fat depth was associated with an increase of 8.3 g (95% CI 2.5, 14.1) in birthweight. The third study^57^ reported VAT thickness as being independently associated with birthweight centile (adjusted *r* ^2^ 15.8%, *p* = 0.002) (Table S6). The fourth study^56^ reported a 1‐cm increase in VAT depth associated with a 1.5 higher birthweight percentile, but the association was not statistically significant (adjusted odds ratio [AOR] 1.5, 95%CI −0.03, 3.00). One study^57^ reported a significant but weak positive correlation with the combination of VAT + SAT (*r* 0.39, *p* = 0.004) (Table S6).

### 4.6 Maternal SFT and birthweight

Two studies^47^, ^73^ reported no significant associations between measures of maternal SFT (triceps, biceps, subscapular, and suprailiac) and birthweight. One^73^ reported negative but no significant association per SD increase in triceps (standardized estimate of −18g, 95% CI −100, 65) or subscapular (−39 g, 95% CI −118, 39) SFT. One^47^ reported positive but non‐significant correlation coefficients for maternal triceps (*r* 0.06), subscapular (*r* 0.09), and suprailiac SFT (*r* 0.15) (*p* value not reported), whereas no correlation was found for maternal biceps SFT (*r* 0, *p* value not reported) (Table S6)

## 5 HIGH BIRTHWEIGHT‐RELATED OUTCOMES

There were 14 studies^23^, ^24^, ^25^, ^28^, ^52^, ^54^, ^55^, ^56^, ^58^, ^61^, ^62^, ^65^, ^66^, ^70^ reporting data for high birthweight‐related outcomes (LGA and macrosomia) and maternal circumference measures (WC and neck circumference), WHR, and fat/mass type (visceral fat, subcutaneous fat, VAT:SAT ratio, FM, and FFM) (Table S7A‐B). Meta‐analysis was possible for maternal WC and macrosomia, and for maternal subcutaneous fat thickness and LGA. Additional data were reported for neck circumference, WHR, and type of fat/mass and high birthweight outcomes that could not be pooled in meta‐analysis. Overall, all adiposity measures showed a pattern towards a positive association with high birthweight outcomes for all maternal adiposity measures, although there were some instances of conflicting data and not all associations were statistically significant. An overview of the meta‐analysis and narrative synthesis for high birthweight are presented for each adiposity exposure.

### 5.1 Maternal WC and high birthweight

**FIGURE 4:**
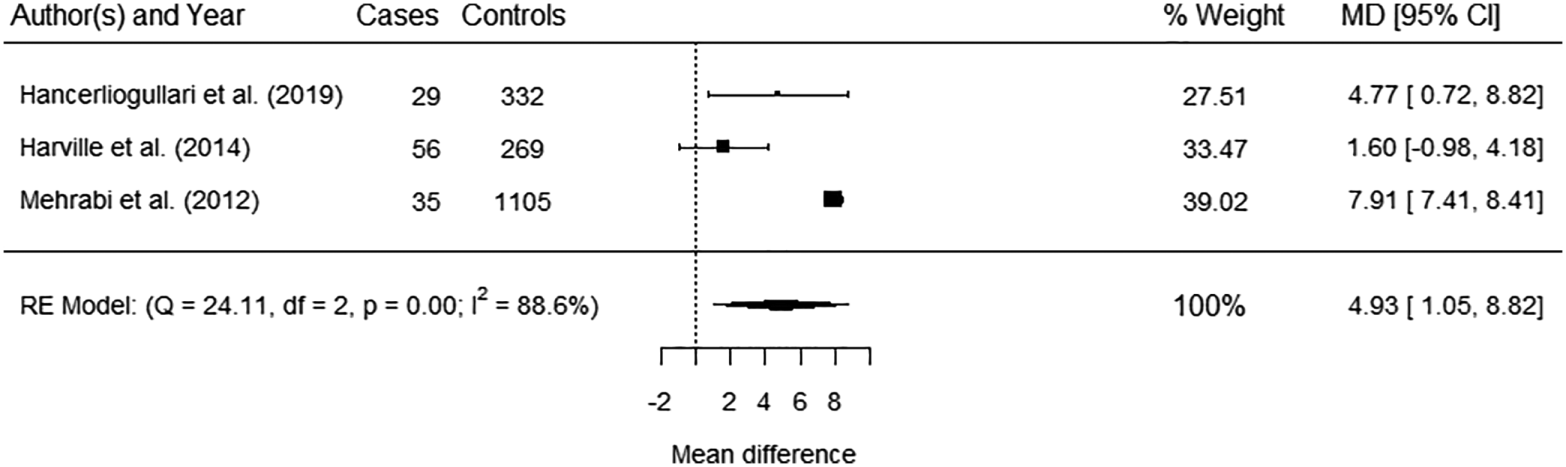
Meta‐analysis of the association between maternal WC (mean differences, cm) and macrosomia. Total sample size (*n* = 1826), MD – mean difference (cm), CI – confidence interval, RE—random effect

Six studies^52^, ^54^, ^55^, ^61^, ^65^, ^70^ reported data for maternal WC. Five^52^, ^54^, ^55^, ^61^, ^70^ reported odds of LGA or macrosomia with continuous or categorical measures of maternal WC but could not be pooled in a meta‐analysis (Table S7A). There was a significant increased odds of macrosomia with maternal WC >88.90 cm  compared with <68.58 cm (AOR 1.58, 95% CI 1.07, 2.32)^61^ and for LGA with maternal WC ≥80 cm compared with <80 cm (AOR 2.14 95% CI 1.21, 3.75).^52^ Two studies^55^, ^70^ reported continuous measures of maternal WC. One^55^ reported significantly increased odds of LGA per SD increase in maternal WC (AOR 1.41, 95% CI 1.00, 1.99) but not for macrosomia (AOR 1.15, 95% CI 0.84, 1.56), and one^70^ showed increased odds of LGA which was not significant (AOR 1.01, 95% CI 0.76, 1.33) (Table S7A). One study^54^ reported an area under the receiving operator curve (AUROC) of 0.63 (95% CI 1.21, 3.75) for maternal WC >88 cm to predict macrosomia (Table S7A). Three studies^54^, ^55^, ^65^ reported case control data for maternal WC and macrosomia that could be pooled in a meta‐analysis (Figure 4), showing significantly increased maternal WC among cases compared with controls (MD 4.93 cm, 95% CI 1.05, 8.82) with significant heterogeneity (*I* ^2^ 88.6%). One study^55^ additionally reported significantly higher maternal WC for LGA cases than controls (Table S7B).

### 5.2 Subcutaneous fat and high birthweight

**FIGURE 5:**
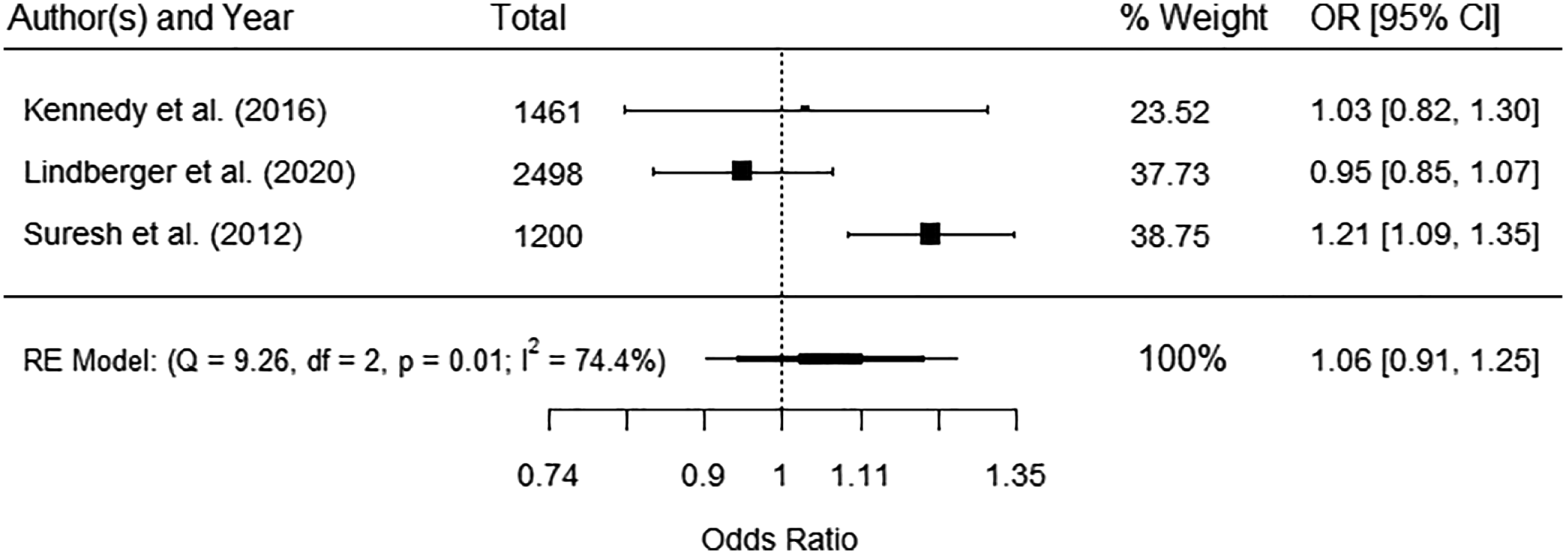
Meta‐analysis of the association between maternal subcutaneous fat (mm) and LGA. Total sample size (*n* = 5159), OR – odds ratio, CI – confidence interval, RE—random effect

There were three studies^23^, ^24^, ^62^ reporting odds of LGA with continuous measures of maternal subcutaneous fat thickness, and all could be pooled in a meta‐analysis (Figure 5) which showed no significant association (OR 1.06 95%CI 0.91, 1.25) with significant heterogeneity (*I* ^2^ 74.4%). One study^24^ additionally reported no significant association between maternal subcutaneous fat and macrosomia (AOR 0.99, 95% CI 0.87, 1.13) (Table S7A).

### 5.3 Maternal neck circumferences and high birthweight

One study^54^ reported an AUROC of 0.65 (95% CI 0.53, 0.75) for maternal neck circumference >36.5 cm to predict macrosomia (Table S7A) and significantly higher median maternal neck circumference for macrosomic infants compared to controls (Table S7B).

### 5.4 Maternal WHR and high birthweight

Four studies^25^, ^28^, ^61^, ^66^ reported data for LGA or macrosomia and continuous or categorical measures of maternal WHR. One^28^ reported significantly increased odds of LGA among those with WHR in the third quartile compared with those with WHR in the first quartile (AOR 1.77, 95% CI 1.09, 2.89) and a trend for increased odds of macrosomia with increasing quartiles of maternal WHR (OR 1.17, 95% CI 1.04,1.31).^28^, ^61^ One^28^ reported significant trends for increased odds of macrosomia (>4.0 kg and >4.5 kg) with increasing maternal WHR quartile (AOR 1.17, 95% CI 1.04, 1.31 and AOR 1.40, 95% CI 1.01, 1.93, respectively). However, when maternal WHR was analyzed according to quartiles, only a significant association was found between third and fourth WHR quartiles and macrosomia >4.0 kg (AOR 1.58, 95% CI 1.10, 2.26 and AOR 1.57, 95% CI 1.07, 2.30, respectively). One study^61^ found a negative but non‐significant association between macrosomia and maternal WHR quintiles (AORs ranging from AOR 0.75, 95% CI 0.55, 1.01 to 1.13, 95% CI 0.85, 1.49). Two studies^25^, ^66^ reported the AUROC data for maternal WHR and LGA; one^25^ reported the AUROC as being 0.514 (*p* = 0.57), and the other^66^ reported 0.713 (*p* not reported but author classified >0.7 as a predictive value).

### 5.5 Types of fat and high birthweight

Three studies^55^, ^56^, ^62^ reported data for maternal visceral fat. One reported significantly increased odds of LGA per 5‐mm increase in maternal visceral fat depth (AOR 1.06, 95%CI 1.02, 1.11),^62^ whereas two found increased odds but no significant association for visceral fat depth and LGA (AOR 1.9, 95% CI 0.8, 4.1)^56^ or maternal visceral fat index and LGA (AOR 1.30, 95% CI 0.98, 1.72) and macrosomia (AOR 1.15, 95% CI 0.87, 1.51)^55^ (Table S7A and B). One study^62^ reported significantly increased odds of LGA per unit increase in the ratio of maternal VAT: SAT (AOR 1.09, 95% CI 1.02, 1.17) (Table S7A). One study^58^ reported significantly increased odds of macrosomia with higher quartile of maternal FFM (AOR ranging from 1.49, 95% CI 1.00, 2.24 to AOR 3.64, 95% CI 2.34, 5.68) but only found significant increased odds of macrosomia with the third quartile of maternal FM (AOR 1.62, 95% CI 1.08, 2.44) (Table S7A).

## 6 LOW BIRTHWEIGHT‐RELATED OUTCOMES

Seven studies^24^, ^56^, ^59^, ^63^, ^66^, ^68^, ^70^ reported data for low birthweight‐related outcomes including birthweight <2500 g, SGA, and IUGR. Maternal adiposity measures were early pregnancy WC, WHR, subcutaneous fat, visceral fat, FM, FFM, and SFT measures. There was a significantly increased odds of birthweight <2500 g with every 5‐mm increase in maternal subcutaneous fat^24^ (AOR 1.22, 95% CI 1.00, 1.47) but not for SGA and maternal WC^70^ (AOR 0.92, 95% CI 0.64, 1.34) or maternal VAT depth^56^ (AOR 0.8, 95% CI 0.4, 1.7), or for IUGR and maternal WHR^59^ (OR 0.08, 95% CI 0.003, 2.27) (Table S8A). One study^66^ reported an AUROC of 0.836 for maternal WC and SGA (>0.7 considered a predictive value). Case control analysis showed significantly lower mean maternal arm circumference,^63^ median FFM, and total body water^68^ for SGA cases compared with controls. However, there was significantly higher sum of maternal skinfolds, and triceps, bicep, and subscapular skinfolds^63^, ^68^ among SGA cases compared with controls. No significant difference in maternal FM was found between SGA cases and controls^68^ (Table S8B).

## 7 FETAL GROWTH AND ANTHROPOMETRY AT BIRTH

### 7.1 Fetal growth

Three studies^36^, ^46^, ^71^ reported data for maternal FM, FFM, and visceral fat (Table S9A). Two^46^, ^71^ reported significant positive correlations (*p* < 0.05) between maternal FM (including total, leg and arm FM in kilogram and percent) and fetal mid‐thigh soft‐tissue measurements at 36 week's gestation,^46^ and change in estimated fetal weight between second and third trimester (standardized β 0.36, SE 9.75, *p* < 0.01)^71^ but not with femur length between second and third trimester (standardized β −0.05, SE 0.01, *p* = 0.88)^71^ (Table S9A). Maternal FFM was positively correlated with fetal mid‐thigh soft tissue at 36 weeks (*p* < 0.05),^46^ fetal head circumference (*r* 0.153, *p* = 0.001), biparietal diameter (*r* 0.124, *p* = 0.003), abdominal circumferences (*r* 0.096, *p* = 0.013), and estimated change in fetal weight per 5 kg change in lean mass (kg) (39.8 g *p* = 0.03) at 35 weeks.^36^ However, there was positive but no correlation with maternal FFM and femoral length at 35 weeks (*r* 0.065, *p* = 0.122), change in estimated fetal weight (standardized β 0.19, SE 15.99, *p* = 0.45),^36^ or negative but no association between maternal FFM and femur length between second and third trimester (standardized β −0.03, SE 0.02, *p* = 0.93).^71^ One study reported a significant positive correlation between maternal visceral fat and fetal mid‐thigh soft tissue at 36 weeks (*p* < 0.01) (Table S9A).

### 7.2 Newborn anthropometry

Four studies^37^, ^49^, ^50^, ^72^ reported data for maternal upper arm circumference, WHR, FM, FFM, and visceral fat. One^72^ reported a significant positive correlation between maternal upper arm circumference and newborn length (*r* 0.238, *p* < 0.005), head circumference (*r* 0.297, *p* < 0.001), and abdominal circumference (*r* 0.226, *p* = 0.003). One^49^ reported that a 0.1 unit increase in maternal WHR significantly predicted newborn birth length (0.2 inches, 95% CI 0.1, 0.4) and head circumference (0.3 cm, 95% CI 0.1, 0.5). One study^37^ reported significant positive correlations between maternal FM and newborn SFT for biceps (*r* 0.040, SE 0.011, *p* < 0.0001), triceps (*r* 0.066, SE 0.015, *p* < 0.0001), suprailiac (*r* 0.064, SE 0.012, *p* < 0.0001), and subscapular (*r* 0.050, SE 0.015, *p* < 0.01), but no significant correlation was found for maternal FFM and newborn SFT (Table S9B). This study also found a significant positive correlation for maternal FFM and newborn head circumference (*r* 0.057, SE 0.020, *p* < 0.01) and crown‐heel length (*r* 0.067, SE 0.029, *p* < 0.05), but not for maternal FM and newborn head, chest, abdominal or mid‐upper arm circumference, or crown‐heel length.^37^ Maternal FM was significantly and positively correlated with infant percent FM measured at 2 weeks old (*r* 0.14, 95% CI 0.07, 0.20) in one study^50^ (Table S9B).

## 8 GESTATIONAL AGE AT DELIVERY

Five studies^23^, ^24^, ^51^, ^52^, ^69^ reported data for maternal WC, visceral fat, and subcutaneous fat (Table S10). There was a significant increased odds of preterm delivery (<37 weeks) with maternal WC ≥88 cm compared with <80 cm (AOR 3.14, 95% CI 1.16, 8.50) but not for maternal WC 80‐88 cm (AOR 1.24, 95% C 0.47, 3.25)^51^ or for mean gestational age and maternal WC ≥80 cm compared with <80 cm (*p* = 0.19).^52^ One study reported an increased risk of preterm delivery with maternal visceral fat ≥5.2 cm compared with <5.2 cm (adjusted relative risk [ARR] 3.1, 95% CI 1.5, 6.5).^69^ Two studies reported conflicting results for maternal subcutaneous fat and preterm birth; one^24^ reported significantly increased odds per 5‐mm increase in maternal subcutaneous fat (AOR 1.23, 95% CI 1.07, 1.44), whereas the other^23^ reported no significant difference between subcutaneous fat ≥5.2 cm and <5.2 cm (AOR 1.00, 95% CI 0.97, 1.03) (Table S10).

## 9 PREGNANCY LOSS

Three studies^53^, ^59^, ^67^ reported data for maternal WC, WHR, self‐reported body shape, visceral fat, FM, and FFM. One^53^ found no significant association with quartiles of maternal WC and spontaneous abortion (adjusted hazard ratio [AHR] ranged between 0.80, 95% CI 0.61, 1.06 and 0.91, 95% CI 0.71, 1.17). Two studies found no significant association between maternal WHR and spontaneous abortion^53^ (AHR ranged between 0.75, 95% CI 0.57, 0.99 and 0.84, 95% CI 0.67, 1.06), missed abortion (OR 1.41, 95% CI 0.16, 12.60), or vesicular mole (OR 0.84, 95% CI 0.03, 21.25).^59^ One study^53^ reported no significant association between maternal self‐reported body shape and missed abortion (AHR ranged between 0.89, 95% CI 0.59, 1.35 and 1.15, 95% CI 0.05, 2.65) (Table S11A). One study^67^ reported a significantly higher mean maternal visceral fat, FM, and FFM for cases of spontaneous abortion among primigravid women compared with controls, but no difference for multigravid women (Table S11A, B).

## 10 NEONATAL MORBIDITY

Two studies^24^, ^59^ reported data for maternal WHR and subcutaneous fat (Table S12). One^24^ found a significant increased odds of neonatal respiratory distress (AOR 1.18, 95% CI 1.0, 1.70) and NICU admission (AOR 1.23, 95% CI 1.07, 1.44) per 5‐mm increase in maternal subcutaneous fat, but not for low Apgar at 1 min (AOR 1.09, 95% CI 0.96, 1.23) or neonatal jaundice (AOR 0.95, 95% CI 0.79, 1.22). One^59^ found increased odds but no significant association between maternal WHR >0.80 compared with ≤0.80 and NICU admission (OR 1.40, 95% CI 0.16, 12.6) (Table S12).

## 11 DISCUSSION

This systematic review has identified a large body of existing evidence from 34 studies, including data from 40,143 pregnancies, which report associations between early pregnancy adiposity measured ≤20 weeks' gestation and infant health‐related outcomes. Maternal early pregnancy WC was the most frequently reported adiposity measure, followed by WHR and measures of FM and FFM. Due to both heterogeneity in reporting, and the limited number of studies reporting data for the same combinations of adiposity exposures and infant outcomes, only four meta‐analyses were performed. These showed a significant association between maternal FFM and birthweight, and for WC and macrosomia, but not for FM and birthweight or subcutaneous fat and LGA. The narrative synthesis of data that could not be included in the meta‐analysis suggests that higher maternal WC was associated with birthweight‐related outcomes, maternal WHR was associated with high birthweight, maternal visceral fat with birthweight and preterm birth, and maternal subcutaneous fat with low birthweight outcomes. While other significant results were observed for different combinations of adiposity exposure and infant outcomes, there was an over‐reliance on data from one or two studies contributing to the narrative synthesis (e.g., fetal growth outcomes), and therefore, results should be interpreted with caution.

This systematic review has strengths and limitations. There are several systematic reviews reporting maternal obesity, measured by BMI, and increased risk of adverse infant health outcomes.^2^, ^3^ To our knowledge, this is the first systematic review and meta‐analysis that explore the use of different early pregnancy maternal adiposity measures that could be used to predict a range of infant health outcomes. Strengths of this review include the rigorous search strategy, supplementary searches, and additional information obtained from authors to maximize the number of studies in the meta‐analyses. The screening, data extraction, and quality assessments were carried out in duplicate to minimize human error and subjectivity. We also aimed to maximize the number of studies we were able to pool in meta‐analysis by transforming the data where appropriate. This was possible only in a limited number of cases, due to the heterogeneity in reporting. For example, there was a variability in the exposure definitions (e.g., adiposity was reported as both continuous measures and by categories). This review also has several limitations. There was a lack of consistency between studies in how outcomes were reported. A range of measures of association were reported, including ORs, correlations, means, medians, and AUROC, with some results from adjusted models and others were univariate analysis. When adjusted models were reported, there was a lack of consistency in the variables included, although maternal age, BMI, parity, behavioral factors (e.g., smoking), and socio‐demographic factors (e.g., ethnic group) were the most consistently included variables. Despite a wealth of existing data, there is a lack of standardized reporting of the adiposity measures across studies limiting the ability to pool results in meta‐analysis. For example, the gestational age when adiposity was measured in the included studies varied and we were not able to explore this in the meta‐regression or sub‐group analysis. Indeed, the small number of studies we were able to include in each meta‐analysis meant we were not able to explore sources of heterogeneity using meta‐regression, sub‐group analysis, publication bias tests, or sensitivity analysis as planned in our protocol.^30^ One way to overcome some of the challenges with heterogeneous reporting is to use individual participant data (IPD) meta‐analysis methods. This would enable a standardized approach to applying definitions to, and analyzing, the data across studies to facilitate direct comparison of adiposity measures to determine which might be best at predicting individual risk.^75^, ^76^ IPD meta‐analysis would also facilitate the direct comparison with BMI, or combining adiposity measures with BMI, in the same population of women.

This research supports the need for early intervention in the prevention of adverse infant‐related risks, starting preconception.^77^ The current evidence base suggests that large‐scale behavioral interventions have been successful at improving maternal behavior^78^ and weight‐related outcomes^78^ but shows limited impact on infant health outcomes.^17^ It has been suggested that this evidence demonstrates that interventions during pregnancy are “too little, too late” to fundamentally improve child health outcomes,^77^ and the preconception period presents a greater opportunity for intervention, based on the life course approach and the embryo development around the time of conception.^77^, ^79^ However, interventions to date have either been universal (i.e., no targeting of high risk groups) or have targeted women based on maternal BMI. As previously discussed, evidence from non‐pregnant populations shows that BMI only identifies half of individuals with adiposity‐related risk.^22^ Similarly, current evidence demonstrates that approximately half of women with an obese BMI have uncomplicated pregnancies and do not require high‐risk care, whereas almost half of women with an overweight BMI develop complications despite not being considered as high risk.^80^ Thus, the (lack of) usefulness of BMI to predict individual risk (and therefore allocation of care/intervention) that is seen in non‐pregnant populations appears to be replicated in pregnancy. Basing decisions to offer additional care on individuals BMI will result in some women receiving additional care without needing it, while others who would benefit from additional care are excluded; therefore, alternatives to BMI need to be explored in the pregnancy context.

This systematic review indicates that there are potential alternative measures of maternal adiposity to BMI that are associated with adverse infant health outcomes, which warrant further investigation to explore whether they better predict risk and could be used to inform targeted intervention approaches. Examples of some potential strong predictor variables include WC, visceral fat, WHR, and FFM. However, the lack of ability to conduct thorough meta‐analysis of all potential adiposity measures and infant health outcomes limits drawing firm conclusions on which specific adiposity measures might be most useful. A number of measures such as WC or WHR have been previously suggested as alternatives to BMI to predict pregnancy related risk as they are more reflective of visceral fat and abdominal adiposity. There is evidence that WC and WHR are largely unaffected until 20 weeks' gestation;^28^, ^81^ thus, the measurement collected in early pregnancy could be a reliable indicator of pre‐ or early‐pregnancy adiposity status. In addition to WC and WHR, findings from this review also suggest the potential use of visceral fat, which could be implemented by incorporating into routine antenatal ultrasound scan appointments.

Further research is required to explore whether a different approach to identifying adiposity‐related risk than current use of BMI, and targeting interventions to women with greatest risk, would result in improved infant outcomes. The strong evidence relating to the fetal environment being important for life‐long health^12^, ^13^, ^14^, ^15^ along with evidence of antenatal interventions in other behavioral fields significantly improving infant health, such as smoking cessation,^17^ suggests that the pregnancy period is an opportunity to improve infant health, along with preconception interventions. Rather than future intervention research repeating diet and physical activity interventions in populations of women above specific BMI thresholds, we should be exploring ways to build on the knowledge we have to optimize effectiveness. Future studies should explore whether adiposity measures could be used in routine care to improve our ability to identify women and infants with the greatest risk, and whether additional clinical care or behavior change interventions are effective at improving infant health outcomes if they are targeted towards women with high adiposity.

## CONFLICTS OF INTEREST

None to declare.

## Supporting information


**TABLE S1** Newcastle‐Ottawa quality assessments (A for cohort and B for case control studies)
**TABLE S2** Contacting authors for additional information
**TABLE S3** Table of included studies/study characteristics
**TABLE S4** Summary of maternal adiposity exposures and fetal and infant outcomes reported
**TABLE S5** Newcastle Ottawa scale for quality assessment (A for cohort studies; B for case control studies)
**TABLE S6** Birthweight
**TABLE S7** High birthweight (A for association data and B for case control data reported)
**TABLE S8** Low birthweight (A for association data and B for case control data reported)
**TABLE S9** Fetal growth and infant anthropometry (A for fetal growth and B for infant anthropometry)
**Table S10** Gestational age at delivery
**Table S11** Pregnancy loss (A for association data and B for case control data reported)
**Table S12** Neonatal morbidity
