# Supplementary material for: Association between maternal adiposity measures and infant health outcomes: A systematic review and meta‐analysis
Source: Obes Rev. 2022 Jul 8;23(10):e13491. doi: 10.1111/obr.13491 (PMC9539955; doi:10.1111/obr.13491)
Supplement: Supplementary file 1 — TABLE S1 Newcastle‐Ottawa quality assessments (A for cohort and B for case control studies) TABLE S2 Contacting authors for additional information TABLE S3 Table of included studies/study characteristics TABLE S4 Summary of maternal adiposity exposures and fetal and infant outcomes reported TABLE S5 Newcastle Ottawa scale for quality assessment (A for cohort studies; B for case control studies) TABLE S6 Birthweight TABLE S7 High birthweight (A for association data and B for case control data reported) TABLE S8 Low birthweight (A for association data and B for case control data reported) TABLE S9 Fetal growth and infant anthropometry (A for fetal growth and B for infant anthropometry) Table S10 Gestational age at delivery Table S11 Pregnancy loss (A for association data and B for case control data reported) Table S12 Neonatal morbidity [file OBR-23-e13491-s001.docx]

**Online Supporting Information**

**Supplementary tables:**

Table S1. Newcastle-Ottawa quality assessments (A for cohort and B for case control studies)

Table S2. Contacting authors for additional information

Table S3. Table of included studies/ study characteristics

Table S4. Summary of maternal adiposity exposures and fetal and infant outcomes reported

Table S5. Newcastle Ottawa scale for quality assessment (A for cohort studies; B for case control studies)

Table S6. Birthweight

Table S7: High birthweight (A for association data and B for case control data reported)

Table S8: Low birthweight (A for association data and B for case control data reported)

Table S9: Fetal growth and infant anthropometry (A for fetal growth and B for infant anthropometry)

Table S10. Gestational age at delivery

Table S11. Pregnancy loss (A for association data and B for case control data reported)

Table S12. Neonatal morbidity

**Table S1A. Newcastle-Ottawa quality assessments for cohort studies ^1^**

| **Selection ^a, b^**  1) Representativeness of the exposed cohort (i.e. those with high adiposity measures) **– select one only**  a) truly representative of the average maternal population in the community (e.g. they recruited all women in a set time period) *  b) somewhat representative of the average maternal population in the community (e.g. they compared the population they recruited with the local population and they have similar characteristics) *****  c) selected group of users eg those with diabetes, over 35, restricted to one ethnic group, PCOS etc  d) no description of the derivation of the cohort  2) Selection of the non-exposed cohort (i.e. those with low adiposity measures) **– select one only**  a) drawn from the same community as the exposed cohort (it will usually be this answer if they have recruited women from the same unit at the same time) *****  b) drawn from a different source (e.g. recruited women from the same unit but at a different time, or from a different unit)  c) no description of the derivation of the non-exposed cohort  3) Ascertainment of exposure **– select one only**  a) secure record (eg explicitly measured adiposity) *****  b) structured interview (e.g. validated self-report such as asking to self-report the pre-pregnancy exposure and then validating this with an in-pregnancy measure – but used the self-report measure in the analysis as the exposure variable) *****  c) any self-report (invalidated)  d) no description  **Comparability**  4) Comparability of cohorts on the basis of the design or analysis - **a and b can both be selected**  a) study controls for change in adiposity in pregnancy (e.g. gestational weight gain) *****  b) study controls for any additional factor *****  c) no factors controlled for  **Outcome**  5) Assessment of outcome **– select one only**  a) independent blind assessment (e.g. specifically measured for the research) *****  b) record linkage (e.g. outcomes from routine medical records) *****  c) self-report  d) no description  6) Was follow-up long enough for outcomes to occur **– select one only**  a) yes (select an adequate follow up period for outcome of interest: followed up for long enough for the outcome to develop, e.g. GDM assessed late in pregnancy, all women followed up until delivery for preterm birth outcome etc) *****  b) no  7) Adequacy of follow up of cohorts **– select one only**  a) complete follow up - all subjects accounted for (i.e. no loss to follow up for prospective cohorts) *****  b) subjects lost to follow up unlikely to introduce bias - small number lost (>80% follow up for prospective cohorts, or for retrospective cohorts >80% with the data required for analysis) or description provided of those lost) *****  c) follow up rate < 80% or >20% excluded due to missing data for the analysis and no description of those lost  d) no statement  Total number of stars (out of a possible 8): |
| --- |

Abbreviation: BMI- Body mass index. a- The exposure is pre- or early pregnancy adiposity (excluding BMI), b- Question 4 “Demonstration that outcome of interest was not present at start of study” in the original scale is not applicable to pregnancy outcomes cannot exist at the start of the study. This item has been removed from the scale and the denominator reduced from 9 to 8.

**Table S1B. Adapted Newcastle-Ottawa Scale for Case-Control Studies**

| Selection ^a^  1) Is the case definition adequate?  a) yes, with independent validation*  b) yes, record linkage or based on self-reports with no reference to primary record  c) no description  2) Representativeness of the cases  a) consecutive or obviously representative series of cases*  b) potential for selection biases or not stated  3) Selection of Controls  a) community controls* (e.g., the same antenatal community (including same hospital/clinics) as cases)  b) Hospital controls (e.g., a different hospital or different clinics within hospitals, etc.)  c) no description  4) Definition of Controls  a) no history of disease (endpoint)*  b) no description of source  Comparability  5) Comparability of cases and controls on the basis of the design or analysis (*both a and b can be selected*)  a) study controls for change in adiposity in pregnancy (e.g. gestational weight gain) *  b) study controls for any additional factor*  c) no factors controlled for  Exposure  6) Ascertainment of exposure  a) secure record (e.g., explicitly measured adiposity)*  b) structured interview where blind to case/control status  c) interview not blinded to case/control status (e.g., adiposity data is collected retrospectively after the outcome case/control status is known)  d) written self-report or medical record only  e) no description  7) Same method of ascertainment for cases and controls  a) yes*  b) no  8) Non-Response rate  a) same rate for both groups*  b) non respondents described  c) rate different and no designation  Total number of stars (out of a possible 9): |
| --- |

Abbreviation: BMI- Body mass index. a- The primary exposure is pre-pregnancy or early pregnancy adiposity measures (excluding BMI)

**Tables S2. Contacting authors for additional information**

| **Citation** | **Reason for contact** | **Author response** | **Data provided** |
| --- | --- | --- | --- |
| Jarvie et al. 2020 ^2^ | To ask for numerical data for associations between visceral adipose tissue and birthweight centiles (BWC). This data is reported in figure 1; however, the paper does not provide numerical data. | Data provided by Dr Dilys Freeman | The data plotted in Fig 1 are unadjusted and adjusted BWC (as described in Fig 1 legend) according to VAT thickness group. Authors also provided detailed results from the stepwise reqression equation statistics. |
| Kent et al. 2013 ^3^ | The 4 quantiles of fat mass and fat-free mass are not defined on the paper | No response |  |
| Mehrabi et al. 2012 ^4^ | No number of cases and controls for waist circumference and macrosomia fetus | Data provided by Dr Mehrabi Esmat | The authors provided the prevalence data for macrosomia among participants: 3.1% |

**Table S3. Characteristics of the included studies**

| **Author, year and country of study** | **Study design and sample size** | **Study period** | **Inclusion criteria** | **Exclusion criteria** | **Maternal adiposity exposures reported** | **Gestation at time of exposure measurement** | **Infant outcomes reported** |
| --- | --- | --- | --- | --- | --- | --- | --- |
| Anglim et al. 2017; Ireland ^5^ | Cohort; n=149 | Jul 2011–Feb 2013 | Singleton, aged >18 years, no history of intrauterine growth restriction or medical problems | Not reported | Fat % and mass; Leg fat % and mass; Arm fat % and mass; Fat-free mass; Visceral fat mass | Booking, gestation not specified | Fetal mid-thigh soft tissue thickness at 28-, 32- and 36-weeks’ gestation |
| Backstrand 1995; Mexico ^6^ | Cohort; n=76 | 1984–1986 | Pregnant mothers infants and children, parents and households of children | Not reported | Arm circumference; Skinfolds (triceps, biceps, subscapular and supra-iliac) | Pre-pregnancy and 1^st^ trimester | Birthweight |
| Balani et al. 2014; UK ^7^ | Case control; n=302 | Not reported | Pregnant women, obese, no known DM | T1/2DM, fasting plasma glucose >95mg/dL, multiple pregnancy, untreated endocrine disturbances, chronic hypertension, PE, medication affecting fasting glucose / insulin | Visceral fat mass | 14–17 weeks | Birthweight |
| Brown et al. 1996; USA ^8^ | Cohort; n=702 | 1989 -1992 | 22-35 years, intending but not attempting pregnancy for 3 months, no contraceptives, no hypertension, renal disease, DM, infertility. | Not reported | Waist:Hip | Preconception measurements | Birthweight; New-born length;  New-born head circumference |
| Diaz et al. 2020; USA ^9^ | Cohort; n=209 | 2011-2014 | Singleton, ≥21 years, BMI 18.5–35 kg/m^2^, 2^nd^ parity, conception without assisted fertility treatments | Medical conditions (e.g. GDM, pregnancy complications), medications influencing fetal growth, smoking, alcohol, athlete. | Fat mass | ± first 10 weeks’ gestation | Infant % fat mass |
| Ebrahimi-Mameghani et al. 2013; Iran ^10^ | Cohort; n=948 | 2009–2010 | Nullip. No: hyperemesis, uterine surgery, recurrent spontaneous abortion, molar pregnancy, chronic disease, special diet | Disproportionate GWG according to initial BMI without preeclampsia or GDM, incomplete delivery file | Waist circumference | 1st trimester | Pre-term birth |
| Gao et al. 2017; China ^11^ | Cohort; n=919 | Jan – Dec 2015 | Live-born babies, no history of mental illnesses or brain diseases | Multiple birth, care initiated >12 weeks’, incomplete data | Waist circumference | <12 weeks | Birthweight; LGA; Gestational age |
| Hahn et al. 2014; Denmark ^12^ | Cohort; n=5132 | 2007–2010 | 18–40 years, stable relationship with male partner, not using fertility treatment, trying to become pregnant | Unable to provide valid e-mail address and their Civil Personal Registration number | Waist circumference; Waist:Hip; Self-reported body shape | Pre-conception | Spontaneous abortion |
| Hancerliogullariminary et al. 2021; Turkey ^13^ | Cohort; n=361 | 2018 | Healthy, 11-13+6 weeks’ gestation, admitted to study Training and Research Hospital | Multip. chronic disease (endocrine disorders, hypertension, rheumatic diseases, thrombophilia), pre-DM | Waist circumference; Neck circumference | 11-13+6 weeks | Birthweight; Macrosomia |
| Harville et al. 2014; Finland ^14^ | Cohort; n=349 | 1983-2007 | Enrolled in the Cardiovascular Risk in Young Finns Study, singleton | T1DM | Waist circumference; Visceral adiposity index | Pre-pregnancy | LGA; Macrosomia |
| Ibrahim et al. 2021; Canada ^15^ | cohort; n=452 | 2012-2014 | Singleton, ≥18 years, no DM, metformin use, PCOS, infertility tx, corticosteroids | Not reported | Visceral adipose tissue depth | 11-14 weeks | Birth weight; LGA; SGA |
| Jarvie et al. 2020; Scotland ^2^ | cohort; n=45 | Not reported | Healthy, normotensive, no significant medical history | Preterm deliveries, missing birthweight data, miscarriage, and pregnancy complications | Waist circumference; Hip circumference; Waist:Hip; Visceral and subcutaneous adipose tissue thickness; VAT+SAT; VAT:SAT | 12 weeks | Birthweight |
| Kennedy et al. 2016; Australia ^16^ | Cohort; n=1461 | 2012-2014 | Pregnant women | Multiple pregnancy, insulin-dependent DM, miscarriage, termination, major anomalies | Abdominal subcutaneous fat thickness | 11–14 weeks | Macrosomia; Low birthweight; Low Apgar 1-min; NICU; pre-term; jaundice; respiratory distress |
| Kent et al. 2013; Ireland ^3^ | Cohort; n=2618 | Jul 2008 –Dec 2011 | Viable singleton, Caucasian | <18 years, unable to give informed consent, pre-existing DM | Fat free mass; Fat mass | 1^st^ trimester | Birthweight; Macrosomia |
| Khare et al. 2017; India ^17^ | Cohort; n=120 | Jan – Dec 2014 | Pregnant, <16 weeks’ gestation | DM, hypertension, cardiac disease, familial dyslipidaemias, hypothyroidism | Waist hip ratio | <16 weeks | Miss abortion; intrauterine growth restriction; NICU admission; Vesicular mole |
| Lacroix et al. 2016; Canada ^18^ | Cohort; n=675 | Jan 2010 –Jul 2013 | Pregnant women, 1st trimester, planning to deliver at the Centre Hospitalier Universitaire de Sherbrooke | <18 years, multiple pregnancy, T1DM/ 1^st^ trimester DM, drugs/alcohol abuse, uncontrolled endocrine disease, major medical conditions | Body fat % | 5–16 weeks | Birth weight |
| Li et al. 2012; USA ^19^ | Cohort; n=6687 | 1995-2003 | Singleton, African-American women, aged 21-69 years (participating in the Black Women's Health Study | Delivered <37 weeks, missing data on BMI, birthweight, or number previous births, pregnant at baseline | Waist circumference; Waist Hip ratio | Pre-pregnancy | Macrosomia |
| Lindberger et al. 2020; Sweden ^20^ | Cohort; n=2261 | 2015-2018 | Singleton, term birth, healthy, 2^nd^ trimester anomaly scan, scan by midwife trained in visceral fat (VF) and subcutaneous fat measurements (SCF) | Missing BMI/birthweight/VF/SCF data, multiple pregnancy, pre-/post-term delivery, miscarriage, GDM, intrauterine fetal death, chronic illness, hypertension, pre-eclampsia | Visceral fat depth; Subcutaneous fat depth;  VF:SCF ratio | 16-19 weeks | Birthweight; LGA |
| Lopez et al. 2011; Argentina ^21^ | Cohort; n=1554 | May 2005 - Dec 2006 | 19–49 years, singleton, gestational age <12 weeks, no clinical symptoms of any concomitant pathology, parity 0–5, non-smokers, smoking <five cigarettes/day, non-alcohol, drinking <20g ethanol/day | Not reported | MUAC; Skinfolds (bicipital, tricipital, subscapular) | <16 weeks | SGA |
| Madhavan et al. 2008; India ^22^ | Cohort; n=106 | Apr 2005 –Apr 2006 | 18-35 years, ≤12 weeks’ gestation at first antenatal visit, singleton, live intrauterine pregnancies | DM, thyroid, pituitary disorders, comorbid conditions/severe systemic illness; metabolic/inherited disorders, drugs causing insulin resistance | Waist circumference; Waist Hip ratio | ≤12 weeks | Birthweight |
| McDonnold et al. 2016; USA ^23^ | Cohort; n=2276 | Jul 2003–Feb 2008 | Low risk, nulliparous, data to determine waist-hip ratio and BMI at enrolment | Congenital anomalies and intrauterine fetal demise | Waist Hip ratio | 9-16 weeks | LGA |
| Mehrabi et al. 2012; Iran ^4^ | Cohort; n=1140 | Not reported | Nulliparous, no disease/recurrent abortions/uterus operations/special diet/hyperemesis, 1^st^ trimester | Withdrawal from study, GWG disproportionate to baseline BMI without having GDM | Waist circumference | 6-10 weeks | Macrosomia |
| Migda et al. 2016; Poland ^24^ | Cohort; n=157 | 2011-2013 | Singleton, early symptoms of metabolic syndrome, Caucasian | Not reported | Waist circumference, Waist Hip ratio | 11-13+6 weeks | LGA; SGA |
| O'Dwyer, et al. 2012; Ireland ^25^ | Cohort; n=3000 | Not reported | Singleton, fetal heart activity present, White European | Had miscarried | Fat mass; Fat free mass,  Body fat %; Visceral fat level | 1^st^ trimester | Spontaneous miscarriage |
| Piuri, et al. 2016; Italy ^26^ | Cohort; n=126 | Jun 2012–Jun 2014 | Pregnant, 1^st^ trimester, outpatient clinic for nuchal translucency / combined test | Twins, existing maternal disease (e.g. DM, hypertension, renal disease) | Fat mass; Fat free mass;  Total body water | 12–14 weeks | SGA |
| Ray et al. 2017; Canada ^27^ | Cohort; n=463 | Aug 2012–May 2015 | Viable singleton, no DM within or outside pregnancy | Not reported | Visceral adipose tissue depth | 11-14 weeks | Pre-term birth |
| Retnakaran et al. 2017; China ^28^ | Cohort; n=1484 | Since Feb 2009 (no end date) | Complete pre-gravid cardio metabolic measurements and delivery data, singleton | >5 weeks pregnant at baseline assessment, weight loss during pregnancy of implausible magnitude | Waist circumference | Pre pregnancy | Birthweight, LGA, SGA |
| Salem et al. 2012; UK (ALSPAC) ^29^ | Cohort; n=3083 | Apr 1991 - Dec 1992 | Nulliparous, singleton, live born, term, documented WHR and birthweight | Not reported | Waist hip ratio | Pre-pregnancy | Macrosomia |
| Suresh et al. 2012; Australia ^30^ | Cohort; n=1200 | 2006-2010 | Nulliparous, non-smoking/drug-using, antenatal ultrasound images of sufficient quality for accurate measurement of SFT | Missing or incomplete data on BMI or outcome measures | Subcutaneous fat tissue | Mean GA 20 weeks | LGA; Pre-term birth |
| Thame et al. 2007; Jamaica *^31^ | Cohort; n=361 | Apr 2003 -Feb 2005 | Singleton, <15 weeks’ gestation, no systemic illness (e.g. Hypertension, DM) or genetic abnormality (e.g. sickle cell) | Not reported | Fat mass; Fat free mass | <15 weeks | Birthweight; new-born anthropometry (head , chest, abdominal, mid-upper arm circumference); crown–heel length; skinfolds (biceps, triceps, suprailiac, subscapular)) |
| Thame et al. 2015; Jamaica *^32^ | Cohort; n=360 | Apr 2003 -Feb 2005 | Singleton, <15 weeks’ gestation, no systemic illness | Multiple pregnancy, >15 weeks’ gestation | Fat free mass | <15 weeks | Birth weight; newborn anthropometry (head circumference, crown–heel length), Fetal measurements at 35 weeks’s gestation (head, abdominal circumference; biparietal diameter; femoral length); Estimated fetal weight at 35 weeks' gestation |
| Toro-Ramos et al. 2016; Brazil ^33^ | Cohort; n=92 | Not reported | Singleton, healthy, non-smoking, at 2^nd^ trimester | Had pre-term babies (<37 weeks’ GA) | Fat mass; Lean body mass | 20 weeks | Birth weight adj. for gestational age; Change in estimated fetal weight and femur length |
| Wibowo et al. 2020; Indonesia ^34^ | Cohort; n=134 | 2017 | Normal delivery, complete data for maternal characteristics and neonatal anthropometry, consent to participate | Chronic disease, preeclampsia, GDM, incomplete data | Upper arm circumference | 1st trimester | Birthweight; Birth length; new-born anthropometry (head, abdominal circumference) |
| Young et al. 2015; Vietnam ^35^ | Trial cohort; n=1436 | Phase 1: Oct-Dec 2011; Phase 2: Mar-May 2012 | 18–40 years, married, living in one of the 20 communes selected, intention to stay in the area for 24 months, planning to have children next year | Pregnant, regularly consumed IFA or MM supplements in the past 2 months, severe anaemia, high-risk pregnancy, haematological diseases | Mid upper arm circumference, muscle and fat; Calf circumference; Skinfolds (triceps, subscapular) | Pre-pregnancy | Birthweight |

Abbreviations: DM- Diabetes Mellitus; GDM – gestational DM; LGA – large for gestational age; SGA – small for gestational age; OGTT- oral glucose tolerance test; GCT- glucose challenge test; GA- gestational Age; SFT- subcutaneous fat thickness, VAT- Visceral adipose tissue thickness; SAT- subcutaneous adipose tissue thickness; VF-visceral fat, SCF-subcutaneous fat; WHR- waist-hip ratio; IFA-iron &folic acid; MM-maternal micronutrient.

* Studies of Thame et al. 2007 and Thame et al. 2015 are from the same cohort, there is an overlap of data on fat free mass and birthweight, new-born crown-heel length and head circumference, so data for these outcomes are presented for Thame et al. 2007.

**Table S4. Summary of maternal adiposity exposures and fetal and infant outcomes reported**

| **Study** | **Exposure** | | | | | | | | | | | | | | | | **Outcome** | | | | | | |
| --- | --- | --- | --- | --- | --- | --- | --- | --- | --- | --- | --- | --- | --- | --- | --- | --- | --- | --- | --- | --- | --- | --- | --- |
|  | **WC** | **HC** | **WHR** | **NC** | **CC** | **AC** | **FM** | **FFM** | **VF** | **SF** | **SFT** | **VAI** | **VAT+SAT** | **Ratio of VF:SF** | **Self-reported body shape** | **Total body water** | **Birthweight** | **Low BW** | **New-born anthropometry** | **High BW** | **Mortality** | **GA** | **Morbidity** |
| Anglim *et al ^5^* |  |  |  |  |  |  | X | X | X |  |  |  |  |  |  |  |  |  | X |  |  |  |  |
| Backstrand *et al ^6^* |  |  |  |  |  | X |  |  |  |  | X |  |  |  |  |  | X |  |  |  |  |  |  |
| Balani *et al ^7^* |  |  |  |  |  |  |  |  | X |  |  |  |  |  |  |  | X |  |  |  |  |  |  |
| Brown *et al ^8^* |  |  | X |  |  |  |  |  |  |  |  |  | X |  |  |  | X |  | X |  |  |  |  |
| Diaz et al ^9^ |  |  |  |  |  |  | X |  |  |  |  |  |  |  |  |  |  |  | X |  |  |  |  |
| Ebrahimi-Mameghani *et al ^10^* | X |  |  |  |  |  |  |  |  |  |  |  |  |  |  |  |  |  |  |  |  | X |  |
| Gao *et al ^11^* | X |  |  |  |  |  |  |  |  |  |  |  |  |  |  |  | X |  |  | X |  | X |  |
| Hahn *et al ^12^* | X |  | X |  |  |  |  |  |  |  |  |  |  |  | X |  |  |  |  |  | X |  |  |
| Hancerliogullariminary *et al ^13^* | X |  |  | X |  |  |  |  |  |  |  |  |  |  |  |  | X |  |  | X |  |  |  |
| Harville *et al ^14^* | X |  |  |  |  |  |  |  |  |  |  | X |  |  |  |  |  |  |  | X |  |  |  |
| Ibrahim *et al ^15^* |  |  |  |  |  |  |  |  | X |  |  |  |  |  |  |  | X | X |  | X |  |  |  |
| Jarvie *et al ^2^* | X | X | X |  |  |  |  |  | X |  |  |  | X | X |  |  | X |  |  |  |  |  |  |
| Kennedy *et al ^16^* |  |  |  |  |  |  |  |  |  | X |  |  |  |  |  |  |  | X |  | X |  | X | X |
| Kent *et al ^3^* |  |  |  |  |  |  | X | X |  |  |  |  |  |  |  |  | X |  |  | X |  |  |  |
| Khare *et al ^17^* |  |  | X |  |  |  |  |  |  |  |  |  |  |  |  |  |  | X |  |  | X |  | X |
| Lacroix *et al ^18^* |  |  |  |  |  |  | X |  |  |  |  |  |  |  |  |  | X |  |  |  |  |  |  |
| Li *et al ^19^* | X |  | X |  |  |  |  |  |  |  |  |  |  |  |  |  |  |  |  | X |  |  |  |
| Lindberger *et al ^20^* |  |  |  |  |  |  |  |  | X | X |  |  |  | X |  |  | X |  |  | X |  |  |  |
| Lopez *et al ^21^* |  |  |  |  |  | X |  |  |  |  | X |  |  |  |  |  |  | X |  |  |  |  |  |
| Madhavan *et al ^22^* | X |  | X |  |  |  |  |  |  |  |  |  |  |  |  |  | X |  |  |  |  |  |  |
| McDonnold *et al ^23^* |  |  | X |  |  |  |  |  |  |  |  |  | X |  |  |  |  |  |  | X |  |  |  |
| Mehrabi *et al ^4^* | X |  |  |  |  |  |  |  |  |  |  |  |  |  |  |  |  |  |  | X |  |  |  |
| Migda *et al ^24^* | X |  | X |  |  |  |  |  |  |  |  |  |  |  |  |  |  | X |  | X |  |  |  |
| O'Dwyer *et al ^25^* |  |  |  |  |  |  | X | X | X |  |  |  |  |  |  |  |  |  |  |  | X |  |  |
| Piuri *et al ^26^* |  |  |  |  |  |  | X | X |  |  |  |  |  |  |  | X |  | X |  |  |  |  |  |
| Ray *et al ^27^* |  |  |  |  |  |  |  |  | X |  |  |  |  |  |  |  |  |  |  |  |  | X |  |
| Retnakaran *et al ^28^* | X |  |  |  |  |  |  |  |  |  |  |  |  |  |  |  | X | X |  | X |  |  |  |
| Salem *et al ^29^* |  |  | X |  |  |  |  |  |  |  |  |  |  |  |  |  |  |  |  | X |  |  |  |
| Suresh *et al ^30^* |  |  |  |  |  |  |  |  |  | X |  |  |  |  |  |  |  |  |  | X |  | X |  |
| Thame *et al ^31^* |  |  |  |  |  |  | X | X |  |  |  |  |  |  |  |  | X |  | X |  |  |  |  |
| Thame *et al ^32^* |  |  |  |  |  |  |  | X |  |  |  |  |  |  |  |  |  |  | X |  |  |  |  |
| Toro-Ramos *et al ^33^* |  |  |  |  |  |  | X | X |  |  |  |  |  |  |  |  | X |  | X |  |  |  |  |
| Wibowo *et al ^34^* |  |  |  |  |  | X |  |  |  |  |  |  |  |  |  |  | X |  | X |  |  |  |  |
| Young *et al ^35^* |  |  |  |  | X | X | X | X |  |  | X |  |  |  |  |  | X |  |  |  |  |  |  |

**WC - Waist circumference, HC - Hip circumference, WHR - Waist: hip ratio, NC - Neck circumference, AC - Arm circumference, CC - calf circumference, FM - Fat mass, FFM - Fat-free mass, VF - Visceral fat, SF - Subcutaneous fat, AT - Adipose tissue, VAT- Visceral adipose tissue thickness, SAT- subcutaneous adipose tissue thickness, SFT – Skinfold thickness, VAI-visceral adiposity index, GA-gestational age at delivery**

**Table S5A. Quality scores for cohort studies (n=33 studies)**

| **Paper** | **Newcastle Ottawa Scale question number and score allocated^a^** | | | | | | | | | |
| --- | --- | --- | --- | --- | --- | --- | --- | --- | --- | --- |
|  | **1** | **2** | **3** | **4** | **5** | **6** | **7** | **Total stars^b^** | **Ranking^c^** | **Reviewers^d^** |
| Anglim et al. 2017 ^5^ | a* | a* | a* | c | a* | a* | d | 5 | Medium | NH and AO |
| Backstrand 1995 ^6^ | a* | a* | a* | c | a* | a* | d | 5 | Medium | LN and LC |
| Brown et al. 1996 ^8^ | b* | a* | a* | a* b* | b* | a* | c | 7 | High | NH and AO |
| Diaz et al. 2020 ^9^ | b* | a* | a* | b* | a* | a* | c | 6 | High | LN and LH |
| Ebrahimi-Mameghani et al. 2013 ^10^ | c | a* | a* | b* | d | a* | a* | 5 | Medium | AO and LC |
| Gao et al. 2017 ^11^ | a* | a* | a* | b* | b* | a* | b* | 7 | High | NH and AO |
| Hahn et al. 2014 ^12^ | b* | a* | c | b* | b* | a* | b* | 6 | High | LH and AO |
| Hancerliogullari et al. 2021 ^13^ | a* | a* | a* | b* | a* | a* | b* | 7 | High | LN and AO |
| Harville et al. 2014 ^14^ | b* | a* | a* | b* | b* | a* | b* | 6 | High | AF and AO |
| Ibrahim et al. 2021 ^15^ | a* | a* | a* | a* | a* | a* | a* | 8 | High | LN and AO |
| Jarvie et al. 2020 ^2^ | b* | c | a* | b* | a* | a* | b* | 6 | High | AF and AO |
| Kennedy et al. 2016 ^16^ | b* | a* | a* | b* | b* | a* | b* | 7 | High | AS and AO |
| Kent et al. 2013 ^3^ | b* | a* | a* | c | b* | a* | a* | 6 | High | LH and AO |
| Khare et al. 2017 ^17^ | b* | c | a* | b* | a* | a* | a* | 6 | High | NH and AO |
| Lacroix et al. 2016 ^18^ | a* | a* | a* | b* | b* | a* | d | 6 | High | NH and AO |
| Li et al. 2012 ^19^ | b* | a* | b* | b* | c | a* | b* | 6 | High | LC and AO |
| Lindberger et al. 2020 ^20^ | a* | a* | a* | b* | a* | a* | a* | 7 | High | LH and AO |
| Lopez et al. 2011 ^21^ | b* | a* | a* | c | b* | a* | d | 5 | Medium | AF and AO |
| Madhava et al. 2008 ^22^ | a* | a* | a* | b* | a* | a* | b* | 7 | High | AS and AO |
| McDonnold et al. 2016 ^23^ | c | a* | d | b* | b* | a* | a* | 5 | Medium | LN and AO |
| Mehrabi et al. 2012 ^4^ | a* | a* | a* | b* | a* | a* | a* | 7 | High | NH and AO |
| Migda et al. 2016 ^24^ | c | c | a* | c | a* | a* | a* | 4 | Medium | LC and AO |
| O'Dwyer, et al. 2012 ^25^ | a* | a* | a* | c | b* | a* | a* | 6 | High | LN and LC |
| Piuri, et al. 2017 ^26^ | a* | a* | a* | c | b* | a* | d | 5 | Medium | NH and AO |
| Ray et al. 2017 ^27^ | b* | a* | a* | b* | d | a* | b* | 6 | High | AO and LC |
| Retnakaran et al. 2017 ^28^ | d | a* | a* | a* b* | a* | a* | a* | 7 | High | AF and AO |
| Salem et al. 2012 ^29^ | d | a* | c | b* | b* | a* | d | 4 | Medium | AS and AO |
| Suresh et al. 2012 ^30^ | a* | a* | a* | b* | a* | a* | a* | 7 | High | LN and AO |
| Thame et al. 2007 ^31^ | b* | a* | b* | c | b* | a* | b* | 6 | High | NH and AO |
| Thame et al. 2015 ^32^ | b* | a* | a* | b* | b* | a* | b* | 7 | High | LC and AO |
| Toro-Ramos et al. 2016 ^33^ | b* | a* | a* | b* | a* | a* | d | 6 | High | AO and LC |
| Wibowo et al. 2020 ^34^ | a* | a* | a* | b* | a* | a* | a* | 7 | High | LN and AO |
| Young et al. 2015 ^35^ | b* | a* | a* | b* | a* | a* | b* | 7 | High | NH and AO |
|  | **n*=33 (84.8%)** | **n*=30 (90.9%)** | **n*=30 (90.9%)** | **n*=27 (81.8%)** | **n*=30 (90.9%)** | **n*=33 (100%)** | **n*=24 (72.7%)** | **Range 5-8** | **Low n=0; Medium n=8, (24.2%); High n=25 (75.8%)** |  |

^a^Newcastle-Ottawa question numbers 1-7, answers and associated number of stars (*) are detailed in Table S4C. ^b^ Minimum number of possible stars to be awarded = 0, maximum number of possible stars to be awarded = 8. ^c^ Categories were allocated as: Low = 0-2 stars, Medium = 3-5 stars, High = 6-8 stars. ^d^ Reviewers: NH: Nicola Heslehurst, AF: Angela Flynn, LN: Lem Ngongalah, LC: Lisa Crowe, AS: Alexandre Simon, AO: Adefisayo Odeniyi

**Table S5B. Quality scores for case-control studies (n=1 study)**

| **Paper** | **Newcastle Ottawa Scale question number and score allocated^a^** | | | | | | | | | | |
| --- | --- | --- | --- | --- | --- | --- | --- | --- | --- | --- | --- |
|  | **1** | **2** | **3** | **4** | **5** | **6** | **7** | **8** | **Total stars ^b^** | **Rating^c^** | **Reviewers ^d^** |
| Balani et al. 2014 ^7^ | a* | a* | a* | a* | b* | a* | a* | a* | 8 | High | LN and LH |
|  | **n*=11 (100%)** | **n*=1 (100%)** | **n*=1 (100%)** | **n*=1 (100%)** | **n*=1 (100%)** | **n*=1 (100%)** | **n*=1 (100%)** | **n*=1 (100%)** |  | **Low or medium n=0; High n=1 (100%)** |  |

^a^ Newcastle-Ottawa question numbers 1-8, answers and associated number of stars (*) are detailed in Table S4D. ^b^ Minimum number of possible stars to be awarded = 0, maximum number of possible stars to be awarded = 9. ^c^ Categories were allocated as: Low = 0-3 stars, Medium = 4-6 stars, High = 7-9 stars. ^d^ Reviewers’: LH: Louise Hayes, LN: Lem Ngongalah

**Table S6. Birthweight**

| **Paper** | **Outcome definition** | **Sample size** | **Reference group** | **Comparison groups** | **Result** | **Adjustments** |
| --- | --- | --- | --- | --- | --- | --- |
| \| **Maternal fat mass (measured by ultra sound)** \| \| \| \| \| \| \| \| --- \| --- \| --- \| --- \| --- \| --- \| --- \| \| Kent et al. 2013 ^3^ \| Birthweight (kg) \| 2618 \| Continuous (kg) \| n/a \| Regression coefficient 0.7 (95% CI -1.9, 3.3) \| Age, parity, gestational age at delivery, smoking, fat free mass \| \| Toro-Ramos et al. 2016 ^33^ \| Birth weight adjusted for gestational age (kg) \| 92 \| Continuous (kg) \| n/a \| **Unstandardized Beta coefficient 22.60 (95%CI 14.35, 30.85)** \| None \| \| Thame et al. 2007 ^31^ \| Birthweight (kg) \| 361 \| Continuous (kg) \| n/a \| B, regression coefficient (r) (3.56 (95%CI -7.24, 14.35) \| None \| \| Lacroix et al. 2016 ^18^ \| Birthweight (g) \| 675 \| Body fat percentage, continuous \| n/a \| **Pearson correlations (r) 0.23, p<0.0001** \| None \| \| Young et al. 2015 ^35^ \| Birth weight (g) \| 1436 \| Continuous \| 1 SD increase in mid upper arm fat area, cm^2^ \| **Standardised estimate 124g (95% CI 73-175)** \| Gestational age, sex of the child, mother’s age, education, treatment group, time from pre-pregnancy enrolment to first prenatal visit and SE \| \| **Maternal fat free mass (measured by ultra sound)** \| \| \| \| \| \| \| \| Kent et al. 2013 ^3^ \| Birthweight (kg) \| 2618 \| Continuous (kg) \| n/a \| **Regression coefficient 19.8 (95% CI 17.0, 22.7), p<0.001** \| Age, parity, gestational age at delivery, smoking, fat mass \| \| Thame et al. 2007 ^31^ \| Birthweight (kg) \| 361 \| Continuous (kg) \| n/a \| **B regression coefficient (r) 12.41 (95%CI 1.61, 23.20)** \| None \| \| Toro-Ramos et al. 2016 ^33^ \| Birth weight adjusted for gestational age (kg) \| 92 \| Continuous (kg) \| n/a \| Unstandardized Beta coefficient 14.50 (95%CI -7.04, 36.04) \| None \| \| Young et al. 2015 ^35^ \| Birth weight (g) \| 1436 \| Continuous \| 1 SD increase in mid upper arm muscle, cm^2^ \| **Standardised estimate 133 (95% CI 82, 183)** \| Gestational age, sex of the child, mother’s age, education, treatment group, time from pre-pregnancy enrolment to first prenatal visit and SE \| | | | | | | |
| **Maternal waist circumference (WC)** | | | | | | |
| Gao et al. 2017 ^11^ | Birthweight (kg) | 919 | <80cm | ≥80cm | **Mean (SD), kg: 3.33 (0.4); 3.51 (0.5), p<0.001** | None |
| Retnakaran et al. 2017 ^28^ | Birthweight (g) | 1484 | Continuous | Per unit (1 cm) change in WC | Adjusted Coefficient 3.5 (95% CI -0.9, 7.9) | Age, education, BMI, systolic BP, LDL & HDL cholesterol, triglycerides, blood glucose, gestation, GWG, GDM, infant sex |
| Madhavan et al. 2008 ^22^ | Birthweight (kg) | 106 | Continuous | n/a | **Correlation (r) 0.6, p<0.01** | None |
| Jarvie et al. 2020 ^2^ | Birthweight centile | 53 | Continuous | n/a | **Correlation (r) 0.42, p<0.05** | None |
| Hancerliogullari et al. 2021 ^13^ | Birthweight (g) | 361 | Continuous | n/a | **Correlation (r) 0.179, p=0.001** | None |
| **Maternal neck circumference** | | | | | | |
| Hancerliogullari et al. 2021 ^13^ | Birthweight (g) | 361 | Continuous | n/a | **Correlation (r) 0.164, p=0.002** | None |
| **Maternal arm circumference** |  |  |  |  |  |  |
| Young et al. 2015 ^35^ | Birthweight (g) | 1436 | Continuous | 1 SD increase in MUAC, cm | Standardised estimate  -5 (95% CI -108 to 97) | Gestational age, sex of the child, mother’s age, education, treatment group, time from pre-pregnancy enrolment to first prenatal visit and SE |
| Backstrand. 1995 ^6^ | Birthweight (g) | 32 | Upper arm circumference, cm, continuous | n/a | Correlation coefficient (r) 0.19, p value not significant | None |
| Wibowo et al. 2020 ^34^ | Birthweight (g) | 134 | Upper arm circumference, cm, continuous | n/a | **Spearman Correlation (r) 0.271, p<0.001** | None |
| **Maternal hip circumference** | | | | | | |
| Jarvie et al. 2020 ^2^ | Birthweight centile | 53 | Continuous | n/a | **Pearson correlation (r) 0.32, p<0.05** | None |
| **Maternal calf circumference** | | | | | | |
| Young et al. 2015 ^35^ | Birthweight (g) | 1436 | Continuous | Per 1 SD increase in calf circumference | Standardised estimate 4 (95% CI -78, 86) | Gestational age, sex of the child, mother’s age, education, treatment group, time from pre-pregnancy enrolment to first prenatal visit and SE |
| **Maternal waist-to-hip ratio** | | | | | | |
| Brown et al. 1996 ^8^ | Birthweight (g) | 508 | Continuous | 0.1 unit increase in WHR | **Parameter Estimates 120 (95% CI 54, 187)** | Income, age, race, education, occupation, parity, BMI, height, skinfold, GWG, prenatal infection, GDM, smoking, infant gestational age and sex |
| Madhavan et al. 2008 ^22^ | Birthweight (kg) | 106 | Continuous | n/a | **Correlation (r) 0.6, p<0.01** | None |
| Jarvie et al. 2020 ^2^ | Birthweight centile | 53 | Continuous | n/a | Pearson correlation (r) 0.16, p=0.25 | None |
| **Maternal ratio of visceral fat: subcutaneous fat (VAT:SAT)** | | | | | | |
| Lindberger et al. 2020 ^20^ | Birthweight (g) | 2498 | Continuous | Per 1 unit increase in VAT:SAT | Adjusted Beta coefficient 7.2 (95%CI -2.4, 16.8), p=0.139 | BMI, age, smoking at first antenatal visit, parity, SCF, country of birth, gestational age and infant sex |
| Jarvie et al. 2020 ^2^ | Birthweight centile | 53 | Continuous | n/a, VAT: SAT | Pearson correlation, (r) 0.01, p=0.97 | None |
| **Maternal subcutaneous fat (SF) measured by ultrasound** | | | | | | |
| Jarvie et al. 2020 ^2^ | Birthweight centile | 53 | Continuous | n/a | **Pearson correlation (r) 0.34, p<0.05** | None |
| Lindberger et al. 2020 ^20^ | Birthweight (g) | 2498 | Continuous | per 5mm increase in SF depth | Adjusted Beta coefficient -0.7 (95%CI -15.4, 13.9) | BMI, age, smoking at first antenatal visit, parity, SCF, country of birth, gestational age and infant sex |
| **Maternal visceral fat (measured by ultrasound unless specified)** | | | | | | |
| Jarvie et al. 2020 ^2^ | Birthweight centile | 53 | Continuous | 1mm increase in VAT thickness, mm | **Adjusted (r^2^) 15.8%, p=0.002** | BMI, waist circumference, hip circumference, SAT thickness, VAT+SAT |
| Lindberger et al. 2020 ^20^ | Birthweight (g) | 2498 | Continuous | Per 5 mm increase in visceral fat depth, mm | **Adjusted β coefficient**  **8.3g (95%CI 2.5, 14.1)** | BMI, age, smoking at first antenatal visit, parity, SCF, country of birth, gestational age and infant sex |
| Ibrahim et al. 2021^15^ | Birthweight percentile | 452 | Continuous | Per 1cm increase in VF depth | AOR 1.5 (95% CI 0.03, 3.0) | Parity, current smoking, change in weight gain from pre-pregnancy to 24-48 weeks |
| Balani et al. 2014 ^7^ | Birth weight (g) | 302 | Continuous | n/a (measured by DSM-BIA)* | **Pearson correlation (r) 0.17, p=0.002** | None |
| **Combination of maternal visceral adipose tissue thickness and subcutaneous adipose tissue thickness (VAT+ SAT)** | | | | | | |
| Jarvie et al. 2020 ^2^ | Birthweight centile | 53 | Continuous | n/a | **Pearson correlation, (r) 0.39, p=0.004** | None |
| **Maternal skinfold thickness (SFT)** | | | | | | |
| Young et al. 2015 ^35^ | Birthweight (g) | 1436 | Continuous | 1 SD increase in triceps SF, mm | Standardised estimate -18 (95%CI -100, 65) | Gestational age, sex of the child, mother’s age, education, treatment group, time from pre-pregnancy enrolment to first prenatal visit, SES |
| Young et al. 2015 ^35^ | Birthweight (g) | 1436 | Continuous | 1 SD increase in subscapular, mm | Standardised estimate -39 (95%CI -118, 39) | Gestational age, sex of the child, mother’s age, education, treatment group, time from pre-pregnancy enrolment to first prenatal visit and SE |
| Backstrand. 1995 ^6^ | Birthweight (g) | 32 | Triceps (1^st^ trimester) | n/a | Correlation coefficient (r) 0.06, NS | None |
| Backstrand. 1995 ^6^ | Birthweight (g) | 32 | Biceps (1^st^ trimester) | n/a | Correlation coefficient (r) 0, NS | None |
| Backstrand. 1995 ^6^ | Birthweight (g) | 32 | Subscapular (1^st^ trimester) | n/a | Correlation coefficient (r) 0.09, NS | None |
| Backstrand. 1995 ^6^ | Birthweight (g) | 32 | Suprailiac (1^st^ trimester) | n/a | Correlation coefficient (r) 0.15, NS | None |

Abbreviation: VAT-Visceral adipose tissue thickness; SCF-subcutaneous fat, SAT- subcutaneous adipose tissue thickness; WC-waist circumference, MUAC-mid-upper arm circumference, NR-not reported; SD-standard deviation; SE-standard error; BMI-body mass index; SES-socio-economic status; VAT: SAT- ratio of visceral adiposity tissue to subcutaneous adiposity tissue

* DSM-BIA - Direct Segmental Multi-frequency Bioelectrical Impedance Analysis

Green highlighted results included in categorical meta-analysis, bold data signify statistically significant results.

**Table S7A. High birthweight (association data)**

| **Paper** | **Outcome definition** | **Sample size** | **Reference group** | **Comparison groups** | **Result** | **Adjustments** |
| --- | --- | --- | --- | --- | --- | --- |
| **Maternal waist circumference (WC)** | | | | | | |
| Li et al. 2012 ^19^ | Macrosomia (BW>4.0 kg) | 5554 | <27 inches | 1: 27-28 inches  2: 29-30 inches  3: 31-34 inches  4: >35 inches | 1: AOR 0.92 (95% CI 0.67, 1.25)  2: AOR 1.13 (95% CI 0.82, 1.54)  3: AOR 1.14 (95% CI 0.81, 1.59)  **4: AOR 1.58 (95% CI 1.07, 2.32)** | Age, questionnaire cycle, marital status, education, income, smoking, parity, BMI |
| Harville et al. 2014 ^14^ | Macrosomia (BW>4000g) | 325 | Continuous | Per 1 SD increase in WC | AOR 1.15 (95% CI 0.84, 1.56) | Age, parity, smoking |
| Hancerliogullari et al. 2021 ^13^ | Macrosomia (BW>4.0 kg) | 361 | <88cm | >88cm | **AUROC 0.63 (95%CI 0.52, 0.72)** | None |
| Retnakaran et al. 2017 ^28^ | LGA (BW for GA >90^th^ percentile) | 1484 | Continuous | Per 1 SD increase in WC | AOR 1.01 (95% CI 0.76, 1.33) | Age, education, BMI, systolic BP, LDL cholesterol, HDL cholesterol, triglycerides, blood glucose, length of gestation, weight gain in pregnancy, gestational diabetes, infant sex |
| Harville et al. 2014 ^14^ | LGA (top 10% for GA) | 325 | Continuous | Per 1 SD increase in WC | **AOR 1.41 (95% CI 1.00, 1.99)** | Age, parity, smoking |
| Gao et al. 2017 ^11^ | LGA (BW >90th percentile, Chinese gender-specific birth weight reference curves) | 919 | <80cm | ≥80cm | **AOR 2.14 (95% CI 1.21, 3.75)** | Street, maternal/paternal age/education/BMI, active or passive smoking, alcohol consumption, family income, parity, infant gender and gestational age |
| **Maternal subcutaneous fat (SF) (measured by ultrasound)** | | | | | | |
| Kennedy et al. 2016 ^16^ | LGA (BW >95^th^ percentile | 1461 | Continuous | Per 5 mm increase SFT | AOR 1.03 (95% CI 0.79, 1.26) | BMI, maternal age, parity, smoking status |
| Suresh et al. 2012 ^30^ | LGA (BW>90^th^ percentile) | 1200 | Continuous | Per 5mm increase in SF | **AOR 1.21 (95% CI 1.09, 1.35)** | maternal age |
| Lindberger et al. 2020 ^20^ | LGA (BW standard deviation score> 90th percentile in the cohort) | 2498 | Continuous | Per 5mm increase in SF depth | AOR 0.95 (95% CI 0.85, 1.07) | Early pregnancy BMI, age, smoking at first antenatal visit, parity, SCF, country of birth, gestational age and infant sex |
| Kennedy et al. 2016 ^16^ | Macrosomia (>4.0 kg) | 1461 | Continuous | Per 5 mm increase | AOR 0.99 (95% CI 0.87, 1.13) | BMI, maternal age, parity, smoking status |
| **Maternal neck circumference** | | | | | | |
| Hancerliogullari et al. 2021 ^13^ | Macrosomia (BW>4.0 kg) | 361 | <36.5 cm | >36.5 cm | **AUROC 0.65 (95% CI 0.53, 0.75)** | None |
| **Maternal waist-to-hip ratio (WHR)** | | | | | | |
| McDonnold et al. 2016 ^23^ | LGA (BW >90% by the Alexander nomogram) | 1687 | <0.80 | 1: 0.80 - 0.84  2: ≥0.85 | 1: AOR 1.33 (95% CI 0.83, 2.13)  2: AOR 1.05 (95% CI 0.67, 1.65) | Age, gestational age at enrolment, years of schooling, race, alcohol, smoking status |
| McDonnold et al. 2016 ^23^ | LGA (BW >90% by the Alexander nomogram) | 1687 | Continuous | n/a | AUROC 0.514, p=0.57 | None |
| Migda et al. 2016 ^24^ | LGA (Birthweight ≥ 95^th^ percentile) | 157 | Continuous | n/a | AUROC 0.713 (authors classify >0.7 as predictive) | None |
| Salem et al. 2012 ^29^ | LGA (BW ≥ 95^th^ percentile adjusted for sex and GA) | 3083 | Q1 (0.67-0.69) | 1: Q2 (0.70-0.72)  2: Q3 (0.74-0.76)  3: Q4 (0.78-0.85) | 1: AOR 1.44 (95% CI 0.86, 2.42)  **2: AOR 1.77 (95% CI 1.09, 2.89)**  3: AOR 1.63 (95% CI 0.97, 2.73) | Age, BMI, smoking status, ethnicity, gestational age |
| Salem et al. 2012 ^29^ | LGA (BW ≥ 95th percentile adjusted for sex and GA) | 3083 | n/a | With increasing WHR quartile | **AOR 1.18 (95% CI 1.01, 1.37)** | Age, BMI, smoking status, ethnicity, gestational age |
| Li et al. 2012 ^19^ | Macrosomia (BW>4.0 kg) | 5388 | <0.72 | 1: 0.72-0.75  2: 0.76-0.79  3: 0.80-0.85  4: ≥0.86 | 1: AOR 0.88 (95% CI 0.67, 1.17)  2: AOR 0.75 (95% CI 0.55, 1.01)  3: AOR 1.07 (95% CI 0.80, 1.42)  4: AOR 1.13 (95% CI 0.85, 1.49) | Age, questionnaire cycle, marital status, education, income, smoking, parity, BMI |
| Salem et al. 2012 ^29^ | Macrosomia (BW>4.0 kg) | 3083 | Q1 (0.67-0.69) | 1: Q2 (0.70-0.72)  2: Q3 (0.74-0.76)  3: Q4 (0.78-0.85) | 1: AOR 1.28 (95% CI 0.87, 1.87)  **2: AOR 1.58 (95% CI 1.10, 2.26)**  **3: AOR 1.57 (95% CI 1.07, 2.30)** | Age, BMI, smoking status, ethnicity, gestational age |
| Salem et al. 2012 ^29^ | Macrosomia (BW>4.0 kg) | 3083 | n/a | With increasing WHR quartile | **AOR 1.17 (95% CI 1.04, 1.31)** | Age, BMI, smoking status, ethnicity, gestational age |
| Salem et al. 2012 ^29^ | Macrosomia (BW≥ 4.5 kg) | 3083 | Q1 (0.67-0.69) | 1: Q2 (0.70-0.72)  2: Q3 (0.74-0.76)  3: Q4 (0.78-0.85) | 1: AOR 1.20 (95% CI 0.37, 3.86)  2: AOR 1.64 (95% CI 0.56, 4.78)  3: AOR 2.63 (95% CI 0.95, 7.26) | Age, BMI, smoking status, ethnicity, gestational age |
| Salem et al. 2012 ^29^ | Macrosomia (BW≥ 4.5 kg) | 3083 | n/a | With increasing WHR quartile | **AOR 1.40 (95% CI 1.01, 1.93)** | Age, BMI, smoking status, ethnicity, gestational age |
| **Maternal ratio of VAT: SAT** | | | | | | |
| Lindberger et al. 2020 ^20^ | LGA (BW standard deviation score> 90th percentile in the cohort) | 2498 | Continuous | Per 1 unit increase | **AOR 1.09 (95% CI 1.02, 1.17)** | BMI, age, smoking at first antenatal visit, parity, SCF, country of birth, gestational age and infant sex |
| **Maternal visceral fat (VF)** **(measured by ultrasound)** | | | | | | |
| Ibrahim et al. 2021 ^15^ | LGA (population-based curve) | 452 | Q1-Q3 (1-4.9cm) | Q4 (4.9-11.4cm) (visceral adiposity tissue depth) | AOR 1.9 (95% CI 0.8-4.1) | Parity, current smoking, change in weight gain from pre-pregnancy to 24-48 weeks |
| Lindberger et al. 2020 ^20^ | LGA (BW standard deviation score> 90th percentile in the cohort) | 2498 | Continuous | Per 5mm increase in VF depth | **AOR 1.06 (95%CI 1.02, 1.11)** | BMI, age, smoking at first antenatal visit, parity, SCF, country of birth, gestational age and infant sex |
| Harville et al. 2014 ^14^ | LGA (BW in the top 10% for GA) | 325 | Continuous | Per 1 SD increase in VAI | AOR 1.30 (95%CI 0.98, 1.72) | Age, parity, smoking |
| Harville et al. 2014 ^14^ | Macrosomia (BW>4.0 kg) | 325 | Continuous | Per 1 SD increase in VAI | AOR 1.15 (95%CI 0.87, 1.51) | Age, parity, smoking |
| **Maternal fat mass** |  |  |  |  |  |  |
| Kent et al. 2013 ^3^ | Macrosomia (BW>4.0 kg) | 2618 | Q1 | Quartiles not defined | 1. AOR 1.47 (95% CI 0.98, 2.19)  **2. AOR 1.62 (95% CI 1.08, 2.44)**  3. AOR 1.42 (95% CI 0.91, 2.23) | Age, parity, gestational age, and smoking history, fat-free mass |
| **Maternal fat free mass** |  |  |  |  |  |  |
| Kent et al. 2013 ^3^ | Macrosomia (BW>4.0 kg) | 2618 | Q1 | Quartiles not defined | **1. AOR 1.49 (95% CI 1.00, 2.24)**  **2. AOR 1.89 (95% CI 1.26, 2.84)**  **3. AOR 3.64 (95% CI 2.34, 5.68)** | Age, parity, gestational age, and smoking history, fat mass |

Abbreviation: BW-birthweight; LGA- large for age; BP-blood pressure; LDL cholesterol, HDL, SF or SCF- subcutaneous fat; AUROC- area under the Receiver operating characteristic; BMI-body mass index; VAT-Visceral adipose tissue thickness; SAT- subcutaneous adipose tissue thickness; SFT- subcutaneous fat thickness, WHR-waist-to-hip ratio; VAI- visceral adiposity index; GA-gestational age, NR-not reported; SD-standard deviation; SE-standard error; Q-quartile

Green highlighted results included in categorical meta-analysis, bold data signify statistically significant results.

**Table S7B. High birthweight (Case control data)**

| **Paper** | **Outcome definition** | **Sample size** | **Data reported** | **Controls (no LGA)** | **Cases (LGA)** | **Significance, p value** |
| --- | --- | --- | --- | --- | --- | --- |
| **Maternal waist circumference** |  |  |  |  |  |  |
| Mehrabi et al. 2012 ^4^ | Macrosomia (BW>4 kg) (primiparous only) | 1140 | Mean WC (SD), cm | **82.14 (0.30)** | **90.05 (1.50)** | **0.0001** |
| Harville et al. 2014 ^14^ | Macrosomia (BW>4.0 kg) | 325 | Mean WC (SD), cm | 77.0 (9.7) | 78.6 (8.8) | 0.27 |
| Hancerliogullari et al. 2021 ^13^ | Macrosomia (BW> 4.0 kg) | 361 | Mean WC (SD), cm | 84.69 (10.02) | 89.46 (10.74) | 0.02 |
| Harville et al. 2014 ^14^ | LGA (BW in the top 10% for GA) | 325 | Mean WC (SD), cm | **76.9 (9.4)** | **80.8 (10.5)** | **0.03** |
| **Maternal neck circumference** | | | | | | |
| Hancerliogullari et al. 2021 ^13^ | Macrosomia (BW> 4.0 kg) | 361 | Median NC (min, max), cm | **36 (30, 50)** | **38 (33, 46)** | **0.015** |
| **Maternal visceral adiposity index (VAI)** | | | | | | |
| Harville et al. 2014 ^14^ | Macrosomia (BW>4.0 kg) | 325 | Mean VAI (SD), cm | 1.5 (1.2) | 1.7 (1.2) | 0.44 |
| Harville et al. 2014 ^14^ | LGA (BW in the top 10% for GA) | 325 | Mean VAI (SD), cm | 1.5 (1.1) | 1.9 (1.6) | 0.20 |

Abbreviation: BW-birthweight; LGA- large for gestational age, BW-birthweight, NC-neck circumference, GA-gestational age, NR-not reported; SD-standard deviation; VAI-visceral adiposity index

Bold data signify statistically significant results.

**Table S8A. Low birthweight (SGA) (including Intra-uterine growth restriction) (association data)**

| **Paper** | **Outcome definition** | **Sample size** | **Reference group** | **Comparison groups** | **Result** | **Adjustments** |
| --- | --- | --- | --- | --- | --- | --- |
| **Maternal waist circumference (WC)** | | | | | | |
| Retnakaran et al. 2017 ^28^ | SGA (BW for GA <10^th^ percentile) | 1484 | Continuous | Per 1 SD increase in WC | AOR 0.92 (95% CI 0.64, 1.34) | Age, education, BMI, systolic BP, LDL & HDL cholesterol, triglycerides, blood glucose, gestational age, GWG, GDM, infant sex |
| Migda et al. 2016 ^24^ | SGA ((BW for GA<10th percentile using age -and sex-specific regional growth chart) | 157 | Continuous | n/a | AUROC 0.836, p value not reported | None |
| **Maternal waist-to-hip ratio** | | | | | | |
| Khare et al. 2017 ^17^ | Intrauterine growth restriction |  | ≤85 | >0.85 | OR 0.08 (95%CI 0.003, 2.27) | None |
| **Maternal subcutaneous fat (SF) measured by ultrasound** | | | | | | |
| Kennedy et al. 2016 ^16^ | Low birthweight (<2500 g) | 1461 | Continuous | Per 5 mm increase | **AOR 1.22 (95% CI 1.00, 1.47)** | BMI, maternal age, parity, smoking status |
| **Maternal visceral fat** | | | | | | |
| Ibrahim et al. 2021 ^15^ | SGA (population-based curve) | 452 | Q1-Q3 (1-4.9cm) | Q4 (4.9-11.4cm) (visceral adiposity tissue depth) | AOR 0.8 (95% CI 0.4, 1.8) | Parity, current smoking, change in weight gain from pre-pregnancy to 24-48 weeks |

Abbreviation: SGA- small for gestational age, BW-birthweight, WC-waist circumference; GA-gestational age, NR-not reported; SD-standard deviation; SE-standard error, Q-quartile, BMI-body mass index, BP-blood pressure, LDL-low density lipid, HDL-high density lipid; AUROC- area under the Receiver operating characteristic.

Blue highlighted results included in categorical meta-analysis, bold data signify statistically significant results.

**Table S8B. Low birthweight (Case-control data)**

| **Paper** | **Outcome definition** | **Sample size** | **Data reported** | **Controls** | **Cases** | **Significance, p value** |
| --- | --- | --- | --- | --- | --- | --- |
| **Maternal arm circumference** | | | | | | |
| Lopez et al. 2011 ^21^ | SGA (BW<3000g) | 1066 | Mean (95% CI) MUAC, cm | **26.2 (25.9, 26.5)** | **24.4 (23.9, 24.9)** | **<0.001** |
| **Maternal fat mass** |  |  |  |  |  |  |
| Piuri et al. 2017 ^26^ | SGA (BW for GA<10th percentile) | 127 | Median (IQR) FM, kg | 15.2 (12.1, 19.1) | 13.0 (12.7–13.5) | NS |
| **Maternal fat free mass** |  |  |  |  |  |  |
| Piuri et al. 2017 ^26^ | SGA (BW for GA<10th percentile) | 127 | Median (IQR) FFM, kg | **43.4 (41.4, 46.7)** | **41.3 (37.7–42.3)** | **p<0.05** |
| Piuri et al. 2017 ^26^ | SGA (BW<10^th^ percentile for GA) | 127 | Median (IQR) TBW, kg | **30.3 (28.9, 32.8)** | **28.1 (26.4–29.8)** | **P<0.05** |
| **Maternal skinfold thickness** |  |  |  |  |  |  |
| Piuri et al. 2017 ^26^ | SGA (BW for GA<10th percentile) | 127 | Median (IQR), sum of triceps, bicep, subscapular, cm | **37 (32, 44)** | **63 (52–76)** | **p<0.001** |
| Lopez et al. 2011 ^21^ | SGA (BW<3000g) | 488 | Mean (95% CI) bicipital skinfold, cm | **8.3 (7.7, 8.9)** | **11.0 (10.5, 11.5)** | **<0.001** |
| Lopez et al. 2011 ^21^ | SGA (BW<3000g) | 488 | Mean (95% CI) tricipital skinfold, cm | **16.1 (15.0, 17.3)** | **20.6 (19.8, 21.4)** | **<0.001** |
| Lopez et al. 2011 ^21^ | SGA (BW<3000g) | 488 | Mean (95% CI) subscapular skinfold, cm | **16.9 (15.8, 17.9)** | **20.7 (19.9, 21.5)** | **<0.001** |

Abbreviation: SGA- small for gestational age, BW-birthweight, GA-gestational age, MUAC-mid-upper arm circumference, FM-fat mass, FFM-fat free mass,TBW-total body water, IQR-inter-quartile range

Bold data signify statistically significant results.

**Table S9A. Fetal growth**

| **Paper** | **Outcome definition** | **Sample size** | **Reference group** | **Comparison groups** | **Result** | **Adjustments** |
| --- | --- | --- | --- | --- | --- | --- |
| **Maternal fat mass** | | | | | | |
| Anglim et al. 2017 ^5^ | Fetal mid-thigh soft-tissue measurements at 36/40 | 149 | Continuous (%) | n/a | **Positive correlation (r not reported), p=0.00** | None |
| Anglim et al. 2017 ^5^ | Fetal mid-thigh soft-tissue measurements at 36/40 | 149 | Continuous (kg) | n/a | **Positive correlation (r not reported), p=0.00** | None |
| Anglim et al. 2017 ^5^ | Fetal mid-thigh soft-tissue measurements at 36/40 | 149 | Continuous Leg fat mass (%) | n/a | **Positive correlation (r not reported), p=0.02** | None |
| Anglim et al. 2017 ^5^ | Fetal mid-thigh soft-tissue measurements at 36/40 | 149 | Continuous Leg fat mass (kg) | n/a | **Positive correlation (r not reported), p=0.00** | None |
| Anglim et al. 2017 ^5^ | Fetal mid-thigh soft-tissue measurements at 36/40 | 149 | Continuous Arm fat mass (%) | n/a | **Positive correlation (r not reported), p=0.10** | None |
| Anglim et al. 2017 ^5^ | Fetal mid-thigh soft-tissue measurements at 36/40 | 149 | Continuous Arm fat mass (kg) | n/a | **Positive correlation (r not reported), p=0.01** | None |
| Toro-Ramos et al. 2016 ^33^ | Change in estimated fetal weight between 2nd and 3rd trimester | 92 | Continuous (%) | n/a | **Standardised Beta co-efficient (SE) 0.36 (9.75), p<0.01** | Energy intake, gender, maternal glucose, parity, height, income |
| Toro-Ramos et al. 2016 ^33^ | Change in femur length between 2nd and 3rd trimester | 92 | Continuous (kg) | n/a | Standardised Beta co-efficient (SE) (association) -0.05 (0.01), p=0.88 | Energy intake, gender, maternal glucose, parity, height, income |
| **Maternal fat free mass (FFM)** | | | | | | |
| Anglim et al. 2017 ^5^ | Fetal mid-thigh soft-tissue measurements at 36/40 | 149 | Continuous (kg) | n/a | **Positive correlation (r not reported), p=0.03** | None |
| Anglim et al. 2017 ^5^ | Fetal mid-thigh soft-tissue measurements at 36/40 | 149 | Continuous Leg muscle (kg) | n/a | **Positive correlation (r not reported), p=0.00** | None |
| 546 Anglim et al. 2017 ^5^ | Fetal l mid-thigh soft-tissue measurements at 36/40 | 149 | Continuous Arm muscle(kg) | n/a | **Positive correlation (r not reported), p=0.00** | None |
| 692 Thame et al. 2015 ^32^ | Fetal head circumference at 35 weeks' gestation (mm) | 348 | Continuous Lean mass (kg) | per 5kg change | **Regression coefficient 0.153, p=0.001 (p value for trend)** | maternal age, parity and socio-economic status |
| Thame et al. 2015 ^32^ | Fetal biparietal diameter 35 weeks' gestation (mm) | 348 | Continuous Lean mass (kg) | per 5kg change | **Regression coefficient 0.124, p=0.003 (p value for trend)** | maternal age, parity and socio-economic status |
| Thame et al. 2015 ^32^ | Fetal abdominal circumference 35 weeks' gestation (mm) | 348 | Continuous Lean mass (kg) | per 5kg change | **Regression coefficient (per 5kg change in lean mass) 0.096, p=0.013 (p value for trend)** | maternal age, parity and socio-economic status |
| Thame et al. 2015 ^32^ | Femoral length at 35 weeks' gestation (mm) | 348 | Continuous Lean mass (kg) | per 5kg change | Regression coefficient 0.065, p=0.122 (p value for trend) | maternal age, parity and socio-economic status |
| Thame et al. 2015 ^32^ | Estimated fetal weight at 35 weeks' gestation (g) | 348 | Continuous Lean mass (kg) | per 5kg change | **Regression coefficient 39.8, p=0.03 (p value for trend)** | maternal age, parity and socio-economic status |
| Toro-Ramos et al. 2016 ^33^ | Change in estimated fetal weight between 2^nd^ and 3^rd^ trimester | 92 | Continuous Lean body mass (kg) | n/a | Standardised Beta coefficient (SE) 0.19 (15.99), p=0.45 | Energy intake, gender, maternal glucose, parity, height, income |
| Toro-Ramos et al. 2016 ^33^ | Change in femur length between 2^nd^ and 3^rd^ trimester | 92 | Continuous Lean body mass (kg) | n/a | Standardised Beta coefficient (SE) -0.03 (0.02), p=0.93 | Energy intake, gender, maternal glucose, parity, height, income |
| **Maternal visceral fat** | | | | | | |
| Anglim et al. 2017 ^5^ | Fetal mid-thigh soft-tissue measurements at 36/40 | 149 | Continuous | n/a | **Positive correlation (r not reported) p=0.00** | None |

Abbreviation: SGA- small for gestational age, BW-birthweight, GA-gestational age, SE-standard error

Bold data signify statistically significant results.

**Table S9B. Infant anthropometry**

| **Paper** | **Outcome definition** | **Sample size** | **Reference group** | **Comparison groups** | **Result** | **Adjustments** |
| --- | --- | --- | --- | --- | --- | --- |
| **Maternal upper arm circumference** | | | | | | |
| Wibowo et al. 2020 ^34^ | Head circumference, cm | 134 | Continuous, cm | n/a | **Spearman Correlation (r) 0.297, p<0.001** | None |
| Wibowo et al. 2020 ^34^ | Birth length, cm | 134 | Continuous, cm | n/a | **Spearman Correlation (r) 0.238, p<0.005** | None |
| Wibowo et al. 2020 ^34^ | Abdominal circumference, cm | 134 | Continuous, cm | n/a | **Spearman Correlation (r) 0.226, p=0.003** | None |
| **Maternal wast-to-hip ratio** | | | | | | |
| Brown et al. 1996 ^8^ | Birth length, inches | 508 | Continuous | 0.1 unit increase in WHR | **Parameter Estimates 0.2 inches (95% CI 0.1, 0.4)** | Income, age, race, education, occupation, parity, BMI, height, skinfold, GWG, prenatal infection, GDM, smoking, infant gestational age and sex |
| Brown et al. 1996 ^8^ | Head circumference, cm | 499 | Continuous | 0.1 unit increase in WHR | **Parameter Estimates 0.3cm (95% CI 0.1, 0.5)** | Income, age, race, education, occupation, parity, BMI, height, skinfold, GWG, prenatal infection, GDM, smoking, infant gestational age and sex |
| **Maternal fat mass** | | | | | | |
| Thame et al. 2007 ^31^ | Head circumference (cm) | 361 | Continuous, kg (estimated by using standard equations) * | n/a | Beta regression coefficient (r)(SEB) -0.020 (0.018), NS | None |
| Thame et al. 2007 ^31^ | Crown–heel length (cm) | 361 | Continuous, kg (estimated by using standard equations) * | n/a | Beta regression coefficient (r) (SEB) -0.017 (0.027), NS | None |
| Thame et al. 2007 ^31^ | Chest circumference (cm) | 361 | Continuous, kg (estimated by using standard equations) * | n/a | Beta regression coefficient (r) (SEB) 0.030 (0.029, NS | None |
| Thame et al. 2007 ^31^ | Abdominal circumference (cm) | 361 | Continuous, kg (estimated by using standard equations) * | n/a | Beta regression coefficient (r) (SEB) 0.022 (0.028), NS | None |
| Thame et al. 2007 ^31^ | Mid-upper arm circumference (cm) | 361 | Continuous, kg (estimated by using standard equations) * | n/a | Beta regression coefficient (r) (SEB) 0.018 (0.012), NS | None |
| Thame et al. 2007 ^31^ | Biceps (mm) | 361 | Continuous, kg (estimated by using standard equations) * | n/a | **Beta regression coefficient (r) (SEB) 0.040 (0.011), p <0.0001** | None |
| Thame et al. 2007 ^31^ | Triceps (mm) | 361 | Continuous, kg (estimated by using standard equations) * | n/a | **Beta regression coefficient (r) (SEB) 0.066 (0.015, p <0.0001** | None |
| Thame et al. 2007 ^31^ | Suprailiac (mm) | 361 | Continuous, kg (estimated by using standard equations) * | n/a | **Beta regression coefficient (r) (SEB) 0.064 (0.012), p <0.0001** | None |
| Thame et al. 2007 ^31^ | Subscapular(mm) | 361 | Continuous, kg (estimated by using standard equations) * | n/a | **Beta regression coefficient (r) (SEB) 0.050 (0.015), p<0.01** | None |
| Diaz et al. 2020 ^9^ | Infant % fat mass measured at 2 weeks old | 209 | Continuous, kg (estimated by using standard equations) * | n/a | **Adjusted β coefficient (r) 0.14 (95% CI 0.07, 0.20)** | Maternal race, new-born sex, GWG, gestational age, delivery mode, Feeding mode at age 2 weeks |
| **Maternal fat free mass** | | | | | | |
| Thame et al. 2007 ^31^ | Head circumference (cm) | 361 | Continuous, kg (estimated by using standard equations) * | n/a | **B, regression coefficient (r) (SEB) 0.057 (0.020), p<0.01** | None |
| Thame et al. 2007 ^31^ | Crown–heel length (cm) | 361 | Continuous, kg (estimated by using standard equations) * | n/a | **B, regression coefficient (r) (SEB) 0.067 (0.029), p<0.05** | None |
| Thame et al. 2007 ^31^ | Chest circumference (cm) | 361 | Continuous, kg (estimated by using standard equations) * | n/a | B, regression coefficient (r) (SEB) 0.041 (0.031), NS | None |
| Thame et al. 2007 ^31^ | Abdominal circumference (cm) | 361 | Continuous, kg (estimated by using standard equations) * | n/a | B, regression coefficient (r) (SEB) 0.040 (0.031), NS | None |
| Thame et al. 2007 ^31^ | Mid-upper arm circumference (cm) | 361 | Continuous, kg (estimated by using standard equations) * | n/a | B, regression coefficient (r) (SEB) 0.009 (0.013), NS | None |
| Thame et al. 2007 ^31^ | Biceps (mm) | 361 | Continuous, kg (estimated by using standard equations) * | n/a | B, regression coefficient (r) (SEB) -0.022 (0.012), NS | None |
| Thame et al. 2007 ^31^ | Triceps (mm) | 361 | Continuous, kg (estimated by using standard equations) * | n/a | B, regression coefficient (r) (SEB) -0.053 (0.016), p<0.01 | None |
| Thame et al. 2007 ^31^ | Suprailiac (mm) | 361 | Continuous, kg (estimated by using standard equations) * | n/a | B, regression coefficient (r) (SEB) -0.040 (0.013), p<0.01 | None |
| Thame et al. 2007 ^31^ | Subscapular(mm) | 361 | Continuous, kg (estimated by using standard equations) * | n/a | B, regression coefficient (r) (SEB) -0.028 (0.016), NS | None |

Abbreviation: BW-birthweight, WHR-waist to hip ratio; GA-gestational age, IQR-inter-quartile range; GDM-gestational diabetes mellitus; SEB-; NS-non significant; GWG-gestational weight gain; NR-not reported; BMI-body mass index

Bold data signify statistically significant results. * Calculations to establish body fat and fat-free mass were performed using standard equations using anthropometric methods such as from skinfold thickness

**Table S10. Gestational age at delivery**

| **Paper** | **Outcome definition** | **Sample size** | **Reference group** | **Comparison groups** | **Result** | **Adjustments** |
| --- | --- | --- | --- | --- | --- | --- |
| **Maternal waist circumference** | | | | | | |
| Ebrahimi-Mameghani et al. 2013 ^10^ | Preterm <37 weeks | 948 | <80cm | 1: 80-88cm  2: >88cm | 1: AOR 1.24 (95% C 0.47, 3.25)  **2: AOR 3.14 (95% CI 1.16, 8.50)** | Preeclampsia, gestational hypertension, GDM, pregnancy delivery |
| Gao et al. 2017 ^11^ | Gestational age (weeks) | 919 | <80cm | ≥80cm | Mean weeks (SDE): 39.1 (1.3); 38.9 (1.9) p=0.19 | None |
| **Maternal visceral fat** | | | | | | |
| Ray et al. 2017 ^27^ | Preterm <37 weeks | 463 | <5.2 cm | ≥5.2 cm | **ARR 3.1 (95% CI 1.5, 6.5)** | Age, parity, chronic hypertension, BMI, ASA use in pregnancy |
| **Maternal subcutaneous fat (SF) (measured by ultra sound)** | | | | | | |
| Suresh et al. 2012 ^30^ | Preterm <37 weeks | 1200 | <5.2 cm | ≥5.2 cm | AOR 1.00 (95% CI 0.97, 1.03) | Maternal age |
| Kennedy et al. 2016 ^16^ | Preterm <37 weeks | 1461 | Continuous | Per 5 mm increase in SFT | **AOR 1.23 (95% CI 1.07, 1.44)** | BMI, maternal age, parity, smoking status |

Abbreviation: BMI-body mass index; ASA-aspirin; SFT-subcutaneous fat thick ness; ARR-adjusted relative risk; BMI-body mass index; GDM-gestational diabetes mellitus

Bold data signify statistically significant results.

**Table S11A. Pregnancy loss (association data)**

| **Paper** | **Outcome definition** | **Sample size** | **Reference group** | **Comparison groups** | **Result** | **Adjustments** |
| --- | --- | --- | --- | --- | --- | --- |
| **Maternal waist circumference** | | | | | | |
| Hahn et al. 2014 ^12^ | Spontaneous abortion | 5132 | <75cm | 1: 75-79cm  2: 80-86cm  3: >87cm | 1: AHR 0.91 (95% CI 0.71, 1.17)  2: AHR 0.80 (95% CI 0.61, 1.06)  3: AHR 0.88 (95% CI 0.57, 1.36) | Age, PA, caffeine, parity, vocational training, education, alcohol, smoking, BMI |
| **Maternal waist-to-hip ratio** | | | | | | |
| Hahn et al. 2014 ^12^ | Spontaneous abortion | 5132 | <0.75 | 1: 0.75-0.79  2: 0.80-0.84  3: ≥0.85 | 1: AHR 0.84 (95% CI 0.67, 1.06)  2: AHR 0.75 (95% CI 0.57, 0.99)  3: AHR 0.78 (95% CI 0.59, 1.03) | Age, PA, caffeine, parity, vocational training, education, alcohol, smoking, BMI |
| Khare et al. 2017 ^17^ | Missed Abortion | 120 | ≤0.80 | >0.80 | OR 1.41 (95% CI 0.16, 12.60) | None |
| Khare et al. 2017 17 | Vesicular mole | 120 | ≤0.80 | >0.80 | OR 0.84 (95%CI 0.03, 21.25) | None |
| **Maternal self-reported body shape** | | | | | | |
| Hahn et al. 2014 ^12^ | Spontaneous abortion | 5132 | Equally all over* | 1. Weight gain on chest/shoulders  2. Weight gain on waist/ stomach  3. Weight gain on hips/ thighs  4. Does not gain weight | 1. AHR 1.15 (95% CI 0.05, 2.65)  2. AHR 1.02 (95% CI 0.85, 1.23)  3. AHR 1.04 (95% CI 0.85, 1.27)  4. AHR 0.89 (95% CI 0.59, 1.35) | Age, physical activity, caffeine consumption, parity, vocational training, education, alcohol consumption, smoking, BMI |

Abbreviation: AHR-Adjusted hazard ratio, PA-physical activity; BMI-body mass index

Bold data signify statistically significant results. * Pre-conception women were asked “When you gain weight, where on your body do you mainly add the weight?”

**Table S11B. Pregnancy loss (Case control data)**

| **Paper** | **Outcome definition** | **Sample size** | **Data reported** | **Controls** | **Cases** | **Significance, p value** |
| --- | --- | --- | --- | --- | --- | --- |
| **Maternal visceral fat** | | | | | | |
| O'Dwyer et al. 2012 ^25^ | Spontaneous miscarriage (primigravidas) | 1482 | Mean visceral fat level (measured by BIA) | 3.3 | **4.2** | **0.005** |
| O'Dwyer et al. 2012 ^25^ | Spontaneous miscarriage (multigravidas) | 1513 | Mean visceral fat level (measured by BIA) | 4.0 | 4.4 | 0.33 |
| **Maternal fat mass** | | | | | | |
| O'Dwyer et al. 2012 ^25^ | Spontaneous miscarriage (primigravidas) | 1482 | Mean fat mass, kg | **21.2** | **24.9** | **0.006** |
| O'Dwyer et al. 2012 ^25^ | Spontaneous miscarriage (multigravidas) | 1513 | Mean fat mass, kg | 22.3 | 24.1 | 0.36 |
| O'Dwyer et al. 2012 ^25^ | Spontaneous miscarriage (primigravidas) | 1482 | Mean body fat, % | 29.8 | 31.4 | 0.11 |
| O'Dwyer et al. 2012 ^25^ | Spontaneous miscarriage (multigravidas) | 1513 | Mean body fat, % | 30.9 | 32.4 | 0.11 |
| **Maternal fat free mass** | | | | | | |
| O'Dwyer et al. 2012 ^25^ | Spontaneous miscarriage (primigravidas) | 1482 | Mean fat free mass, kg | **45.3** | **47.0** | **0.04** |
| O'Dwyer et al. 2012 ^25^ | Spontaneous miscarriage (multigravidas) | 1513 | Mean fat free mass, kg | 47.6 | 46.8 | 0.35 |

Abbreviation: AHR- Adjusted hazard Ratio; BIA- Bioelectrical Impedance Analysis.

Bold data signify statistically significant results.

**Table S12. Neonatal morbidity**

| **Paper** | **Outcome definition** | **Sample size** | **Reference group** | **Comparison groups** | **Result** | **Adjustments** |
| --- | --- | --- | --- | --- | --- | --- |
| **Maternal waist-to-hip ratio** | | | | | | |
| Khare et al. 2017**^17^** | NICU admission | 120 | ≤0.80 | >0.80 | OR 1.40 (95% CI 0.16, 12.6) | None |
| **Maternal subcutaneous fat (measured by ultrasound)** | | | | | | |
| Kennedy et al. 2016**^16^** | Low Apgar at 1 minute | 1461 | Continuous | Per 5 mm increase | AOR 1.09 (95% CI 0.96, 1.23) | BMI, maternal age, parity, smoking status |
| Kennedy et al. 2016**^16^** | Neonatal jaundice | 1461 | Continuous | Per 5 mm increase | AOR 0.95 (95% CI 0.79, 1.22) | BMI, maternal age, parity, smoking status |
| Kennedy et al. 2016**^16^** | Neonatal respiratory distress | 1461 | Continuous | Per 5 mm increase | **AOR 1.18 (95% CI 1.0, 1.70)** | BMI, maternal age, parity, smoking status |
| Kennedy et al. 2016**^16^** | NICU admission | 1461 | Continuous | Per 5 mm increase | **AOR 1.23 (95% CI 1.07, 1.44)** | BMI, maternal age, parity, smoking status |

Abbreviation: NICU- new-born intensive care unit; SFT-subcutaneous fat thickness; BMI-body mass index

Bold data signify statistically significant results.

**Supporting information references**

1. Wells GA, Shea B, O’Connell D, et al. The Newcastle-Ottawa Scale (NOS) for assessing the quality of nonrandomised studies in meta-analyses. Oxford; 2000.

2. Jarvie EM, Stewart FM, Ramsay JE, et al. Maternal Adipose Tissue Expansion, A Missing Link in the Prediction of Birth Weight Centile. *J Clin Endocrinol Metab*. Mar 1 2020;105(3)doi:10.1210/clinem/dgz248

3. Kent E, O'Dwyer V, Fattah C, Farah N, O'Connor C, Turner MJ. Correlation between birth weight and maternal body composition. *Obstet Gynecol*. Jan 2013;121(1):46-50. doi:<http://10.1097/AOG.0b013e31827a0052>

4. Mehrabi E, Kamalifard M, Yavarikia P, Ebrahimi Mameghani M. The Relation between Early Pregnancy Anthropometric Indices among Primiparous Women and Macrosomia. *J Caring Sci*. Sep 2012;1(3):153-8. doi:10.5681/jcs.2012.022

5. Anglim B, Farah N, O'Connor C, Daly N, Kennelly MM, Turner MJ. The relationship between maternal body composition in early pregnancy and foetal mid-thigh soft-tissue thickness in the third trimester in a high-risk obstetric population. *J Obstet Gynaecol*. Jul 2017;37(5):591-594. doi:10.1080/01443615.2017.1283303

6. Backstrand JR. Annex: Maternal anthropometry as a risk predictor of pregnancy outcome: the Nutrition CRSP in Mexico. *Bull World Health Organ*. 1995;73 Suppl(Suppl):96-98.

7. Balani J, Hyer S, Johnson A, Shehata H. The importance of visceral fat mass in obese pregnant women and relation with pregnancy outcomes. *Obstet Med*. Mar 2014;7(1):22-5. doi:10.1177/1753495X13495192

8. Brown JE, Potter JD, Jacobs DR, Jr., et al. Maternal waist-to-hip ratio as a predictor of newborn size: Results of the Diana Project. *Epidemiology*. Jan 1996;7(1):62-6. doi:10.1097/00001648-199601000-00011

9. Diaz EC, Cleves MA, DiCarlo M, et al. Parental adiposity differentially associates with newborn body composition. *Pediatr Obes*. Apr 2020;15(4):e12596. doi:10.1111/ijpo.12596

10. Ebrahimi-Mameghani M, Mehrabi E, Kamalifard M, Yavarikia P. Correlation between Body Mass Index and Central Adiposity with Pregnancy Complications in Pregnant Women. *Health Promot Perspect*. 2013;3(1):73-9. doi:10.5681/hpp.2013.009

11. Gao X, Yan Y, Xiang S, et al. The mutual effect of pre-pregnancy body mass index, waist circumference and gestational weight gain on obesity-related adverse pregnancy outcomes: A birth cohort study. *PLoS One*. 2017;12(6):e0177418. doi:10.1371/journal.pone.0177418

12. Hahn KA, Hatch EE, Rothman KJ, et al. Body size and risk of spontaneous abortion among danish pregnancy planners. *Paediatr Perinat Epidemiol*. Sep 2014;28(5):412-23. doi:10.1111/ppe.12142

13. Hancerliogullari N, Kansu-Celik H, Asli Oskovi Kaplan Z, Oksuzoglu A, Ozgu-Erdinc AS, Engin-Ustun Y. Correlation of Maternal Neck/Waist Circumferences and Fetal Macrosomia in Low-Risk Turkish Pregnant Women, a Preliminary Study. *Fetal Pediatr Pathol*. Jun 2021;40(3):181-188. doi:10.1080/15513815.2019.1675831

14. Harville EW, Juonala M, Viikari JS, Raitakari OT. Preconception metabolic indicators predict gestational diabetes and offspring birthweight. *Gynecol Endocrinol*. Nov 2014;30(11):840-4. doi:10.3109/09513590.2014.937336

15. Ibrahim YA, Park AL, Berger H, Ray JG. Maternal Visceral Adipose Tissue and Risk of Having a Small or Large for Gestational Age Infant. *J Obstet Gynaecol Can*. Aug 2021;43(8):973-977. doi:10.1016/j.jogc.2020.11.019

16. Kennedy NJ, Peek MJ, Quinton AE, et al. Maternal abdominal subcutaneous fat thickness as a predictor for adverse pregnancy outcome: a longitudinal cohort study. *BJOG*. Jan 2016;123(2):225-32. doi:10.1111/1471-0528.13758

17. Khare D, Modi J. Waist hip ratio in early pregnancy as a clinical indicator of serum lipid levels and predictor of pregnancy complications. *International Journal of Reproduction, Contraception, Obstetrics and Gynecology*. 2016:1709-1713. doi:10.18203/2320-1770.ijrcog20161444

18. Lacroix M, Battista MC, Doyon M, et al. Higher maternal leptin levels at second trimester are associated with subsequent greater gestational weight gain in late pregnancy. *BMC Pregnancy Childbirth*. Mar 22 2016;16:62. doi:10.1186/s12884-016-0842-y

19. Li S, Rosenberg L, Palmer JR, Phillips GS, Heffner LJ, Wise LA. Central Adiposity and Other Anthropometric Factors in Relation to Risk of Macrosomia in an African American Population. *Obesity*. 2012;21(1):178-184. doi:10.1038/oby.2012.142

20. Lindberger E, Wikstrom AK, Bergman E, et al. Association of maternal central adiposity measured by ultrasound in early mid pregnancy with infant birth size. *Sci Rep*. Nov 12 2020;10(1):19702. doi:10.1038/s41598-020-76741-8

21. Lopez LB, Calvo EB, Poy MS, del Valle Balmaceda Y, Camera K. Changes in skinfolds and mid-upper arm circumference during pregnancy in Argentine women. *Matern Child Nutr*. Jul 2011;7(3):253-62. doi:10.1111/j.1740-8709.2009.00237.x

22. Madhavan A, Beena Kumari R, Sanal MG. A pilot study on the usefulness of body mass index and waist hip ratio as a predictive tool for gestational diabetes in Asian Indians. *Gynecol Endocrinol*. Dec 2008;24(12):701-7. doi:10.1080/09513590802444134

23. McDonnold M, Mele LM, Myatt L, et al. Waist-to-Hip Ratio versus Body Mass Index as Predictor of Obesity-Related Pregnancy Outcomes. *Am J Perinatol*. May 2016;33(6):618-24. doi:10.1055/s-0035-1569986

24. Migda M, Migda MS, Migda B, Krzyzanowska P, Wender-Ozegowska E. Components of metabolic syndrome in the first trimester of pregnancy as predictors of adverse perinatal outcome. *Ginekol Pol*. 2016;87(9):644-650. doi:10.5603/GP.2016.0060

25. O'Dwyer V, Monaghan B, Fattah C, Farah N, Kennelly MM, Turner MJ. Miscarriage after sonographic confirmation of an ongoing pregnancy in women with moderate and severe obesity. *Obes Facts*. 2012;5(3):393-8. doi:10.1159/000336253

26. Piuri G, Ferrazzi E, Bulfoni C, Mastricci L, Di Martino D, Speciani AF. Longitudinal changes and correlations of bioimpedance and anthropometric measurements in pregnancy: Simple possible bed-side tools to assess pregnancy evolution. *J Matern Fetal Neonatal Med*. Dec 2017;30(23):2824-2830. doi:10.1080/14767058.2016.1265929

27. Ray JG, De Souza LR, Park AL, Connelly PW, Bujold E, Berger H. Preeclampsia and Preterm Birth Associated With Visceral Adiposity in Early Pregnancy. *J Obstet Gynaecol Can*. Feb 2017;39(2):78-81. doi:10.1016/j.jogc.2016.10.007

28. Retnakaran R, Wen SW, Tan H, et al. Maternal pre-gravid cardiometabolic health and infant birthweight: A prospective pre-conception cohort study. *Nutr Metab Cardiovasc Dis*. Aug 2017;27(8):723-730. doi:10.1016/j.numecd.2017.05.005

29. Salem W, Adler AI, Lee C, Smith G. Maternal waist to hip ratio is a risk factor for macrosomia. *BJOG : an international journal of obstetrics and gynaecology*. 02/01 2012;119:291-7. doi:10.1111/j.1471-0528.2011.03167.x

30. Suresh A, Liu A, Poulton A, et al. Comparison of maternal abdominal subcutaneous fat thickness and body mass index as markers for pregnancy outcomes: A stratified cohort study. *Aust N Z J Obstet Gynaecol*. Oct 2012;52(5):420-6. doi:10.1111/j.1479-828X.2012.01471.x

31. Thame M, Trotman H, Osmond C, Fletcher H, Antoine M. Body composition in pregnancies of adolescents and mature women and the relationship to birth anthropometry. *Eur J Clin Nutr*. Jan 2007;61(1):47-53. doi:10.1038/sj.ejcn.1602484

32. Thame M, Osmond C, Trotman H. Fetal growth and birth size is associated with maternal anthropometry and body composition. *Matern Child Nutr*. Oct 2015;11(4):574-82. doi:10.1111/mcn.12027

33. Toro-Ramos T, Sichieri R, Hoffman DJ. Maternal fat mass at mid-pregnancy and birth weight in Brazilian women. *Ann Hum Biol*. May 2016;43(3):212-8. doi:10.3109/03014460.2015.1032348

34. Wibowo N, Irwinda R, Rachman L. First trimester maternal upper arm circumference correlated to placental size and neonatal anthropometry. *Medical Journal of Indonesia*. 2020;29(1):38-41. doi:10.13181/mji.oa.192950

35. Young MF, Nguyen PH, Addo OY, et al. The relative influence of maternal nutritional status before and during pregnancy on birth outcomes in Vietnam. *Eur J Obstet Gynecol Reprod Biol*. Nov 2015;194:223-7. doi:10.1016/j.ejogrb.2015.09.018
